# Vaccine plus microbicide effective in preventing vaginal SIV transmission in macaques

**DOI:** 10.1038/s41564-023-01353-7

**Published:** 2023-04-06

**Authors:** Mohammad Arif Rahman, Massimiliano Bissa, Isabela Silva de Castro, Sabrina Helmold Hait, James D. Stamos, Farzana Bhuyan, Ruth Hunegnaw, Sarkis Sarkis, Anna Gutowska, Melvin N. Doster, Ramona Moles, Tanya Hoang, Lisa M. Miller Jenkins, Ettore Appella, David J. Venzon, Hyoyoung Choo-Wosoba, Timothy Cardozo, Marc M. Baum, Daniel H. Appella, Marjorie Robert-Guroff, Genoveffa Franchini

**Affiliations:** 1 Animal Models and Retroviral Vaccines Section, National Cancer Institute, Bethesda, MD USA; 2 Section on Immune Biology of Retroviral Infection, National Cancer Institute, Bethesda, MD USA; 3 Translational Autoinflammatory Diseases Section, Laboratory of Clinical Immunology and Microbiology, National Institute of Allergy and Infectious Diseases, National Institutes of Health, Bethesda, MD USA; 4 Collaborative Protein Technology Resource, Laboratory of Cell Biology, National Cancer Institute, Bethesda, MD USA; 5 Chemical Immunology Section, National Cancer Institute, Bethesda, MD USA; 6 Biostatistics and Data Management Section, Center for Cancer Research, National Cancer Institute, Bethesda, MD USA; 7 New York University School of Medicine, NYU Langone Health, New York, NY USA; 8 Oak Crest Institute of Science, Monrovia, CA USA; 9 Synthetic Bioactive Molecules Section, National Institute of Diabetes and Digestive and Kidney Diseases, National Institutes of Health, Bethesda, MD USA

**Keywords:** HIV infections, Vaccines, Mucosal immunology, Innate immunity

## Abstract

The human immunodeficiency virus epidemic continues in sub-Saharan Africa, and particularly affects adolescent girls and women who have limited access to antiretroviral therapy. Here we report that the risk of vaginal simian immunodeficiency virus (SIV)_mac251_ acquisition is reduced by more than 90% using a combination of a vaccine comprising V1-deleted (V2 enhanced) SIV envelope immunogens with topical treatment of the zinc-finger inhibitor SAMT-247. Following 14 weekly intravaginal exposures to the highly pathogenic SIV_mac251_, 80% of a cohort of 20 macaques vaccinated and treated with SAMT-247 remained uninfected. In an arm of 18 vaccinated-only animals without microbicide, 40% of macaques remained uninfected. The combined SAMT-247/vaccine regimen was significantly more effective than vaccination alone. By analysing immune correlates of protection, we show that, by increasing zinc availability, SAMT-247 increases natural killer cytotoxicity and monocyte efferocytosis, and decreases T-cell activation to augment vaccine-induced protection.

## Abstract (web summary)

Topical microbicide combined with vaccine effectively prevents infection of female macaques with simian immunodeficiency virus.

## Main

Although available treatments have reduced new HIV infections from the peak level of 3.2 million recorded in 1996 by 54% in 2021, infection with human immunodeficiency virus (HIV) continues to cause substantial morbidity and mortality. An estimated 1.5 million people worldwide acquired HIV in 2021 (ref. ^1^). Women are not only biologically more susceptible to HIV-1 infection than men, but they are often socially and culturally more vulnerable to infection as well^1^. Approximately 5,000 women aged 15–24 years are infected with HIV every week globally, and in sub-Saharan Africa six out of seven new HIV infections in adolescents occur in girls^1,2^. An effective HIV vaccine is urgently needed, and specifically one to reduce the burden of disease in women and curb the HIV epidemic.

Of the nine clinical HIV vaccine efficacy trials carried out in humans so far^3–12^, only the RV144 canarypox-based (ALVAC) vaccine demonstrated a modest degree of equivalent efficacy in males and females^8^. The ALVAC-based/gp120/alum vaccine platform, which encodes virus-like particles, reduced virus acquisition by 31.2% in humans in a phase III efficacy trial^8^.

The macaque model has convincingly demonstrated the pre-clinical potential of the ALVAC/simian immunodeficiency virus (SIV) vaccine modality boosted with gp120 (ref. ^13^) by reproducing the efficacy of the successful RV144 HIV vaccine trial testing the alum adjuvant^8^ and predicting the failure of the HVTN-702 trial in South Africa, using the MF59 adjuvant^10^. Moreover, work in this model has demonstrated that the efficacy of ALVAC-based HIV vaccine candidates can be improved by using a DNA prime^14^, simplifying the vaccine regimen^15^, and by better exposing the α-helical conformation of variable region 2 (V2) via the deletion of V1 (ref. ^16^). In vaccinated macaques, V2-specific ADCC correlated with decreased risk of virus acquisition^16^ consistent with the primary and secondary correlates of risk in RV144 (ref. ^17^). Both human and macaque antibodies recognizing the α-helical conformation of V2 inhibit V2-mediated CD4^+^ T-cell co-stimulation and CCR5 expression^18^. Systems biology and functional analyses of CD14^+^ cells in vaccinated macaques demonstrated that the efficacy of the improved DNA/ALVAC gp120/alum platform results from the engagement of the CCR2/CCL2 anti-inflammatory axis^15,19^, activation of the c-AMP/CREB1 pathway in anti-inflammatory (M2-like) monocytes^20^ and monocyte efferocytosis^15,21^ necessary for the clearance of apoptotic cells^22^. In addition, in macaques the vaccine-induced recruitment of NKp44 IL-17^+^ cells to mucosal sites and the decreased expression of CCR5 on Th1 and Th2 cells are also correlates of decreased risk of virus acquisition^13,14,23^.

Thus, while the main immune correlates of risk are increasingly understood, the efficacy of the ∆V1DNA/ALVAC/∆V1gp120/alum vaccine regimen remains suboptimal in both female and male macaques, decreasing the per/exposure risk of virus acquisition by an average of 70%, and protecting approximately half of vaccinated animals from SIV_mac251_ acquisition^15^. The SAMT-247 (S-acyl-2-mercaptobenzamide thioester) microbicide notably targets the mutationally intolerant HIV nucleocapsid (NC) protein zinc fingers^24^ and, by acetylating the cysteine sidechains of the NC protein, causes zinc ejection and loss of virus infectivity^25,26^. Importantly, SAMT-247 exhibits an intracellular recycling mechanism whereby released thiol is re-acetylated by acetyl-CoA, resulting in generation of a reactive thioester compound, thus allowing for numerous rounds of virucidal activity^27^.

The SAMT class of compounds has prevented HIV transmission in vitro in cell-based assays, in explant cultures and to varying degrees in transgenic mice and macaques^28–31^. In this Article, we evaluated whether the SAMT microbicide could increase the potency of an RV144-like vaccine in macaques and its potential suitability for use in human trials, as no other vaccine platforms has yet been clinically effective in humans^3–12^.

We observed a substantial synergy between the microbicide and the engineered V1-deleted envelope immunogens, delivered by the DNA/ALVAC vaccine platform^15,16^; the combined approach reduced the risk of SIV infection in female macaques by more than 90%. SAMT-247, a zinc finger protein inhibitor with virucidal activity, unexpectedly also augmented vaccine-induced protective responses, probably by increasing zinc availability. We propose that our pre-clinical study charts a viable path towards effectively preventing HIV transmission in women.

## Results

### Mucosal microbicide reduces SIV_mac251_ acquisition

**Fig. 1:**
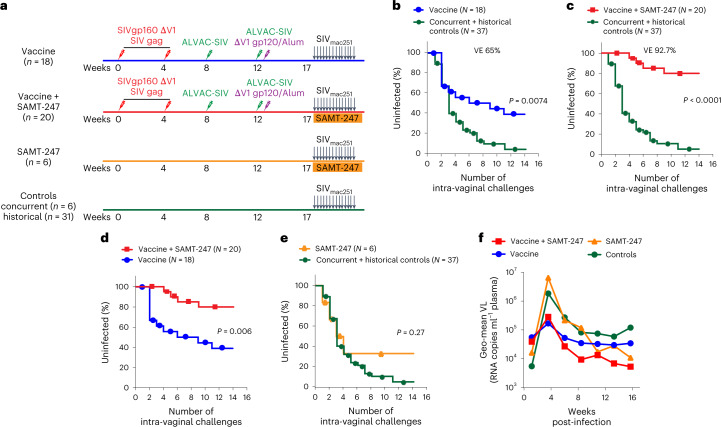
Immunization regimen, infection rate and SIV plasma virus. **a**, Rhesus macaques were subdivided into four groups: vaccine (*n* = 18), vaccine + SAMT-247 (*n* = 20), SAMT-247 (*n* = 6), and concurrent and historical controls (*n* = 6 and 31). Thirty-eight animals were primed with ΔV1 DNA-SIVgp160+p57 Gag and boosted with ALVAC-SIV encoding *env*, *gag* and *pol* and ALVAC-SIV + ΔV1 gp120 protein in alum hydroxide at the indicated timepoints. Twelve animals remained naïve until SIV challenge. Beginning at week 17, vaccine efficacy (VE) was assessed by subjecting all animals to up to 14 weekly intravaginal viral exposures (arrows) in the presence or absence of SAMT-247 until infection was confirmed. Animals either received 0.8% SAMT-247 in HEC gel (*n* = 26) or HEC gel only (*n* = 24) 4 h before each low-dose SIV_mac251_ challenge. **b**,**c**, Significant protection in the vaccine group (*P* = 0.0074) (**b**) and the vaccine + SAMT-247 group (*P* < 0.0001) (**c**) compared with concurrent + historical controls. **d**, Delayed SIV acquisition in the vaccine + SAMT-247 group compared with the vaccine-only group (*P* = 0.006). **e**, No differences in delayed acquisition in the SAMT-247 group were observed compared with the combined concurrent plus historical controls (*P* = 0.27). **f**, Viral load (VL) geometric means of all macaque groups over time. Productive infection was qualified by the presence of viral DNA and RNA in mucosa and persistence of viral RNA in plasma over time. Data shown in **b**–**e** were analysed with log-rank (Mantel–Cox) test. Source data

**Extended Data Fig. 1:**
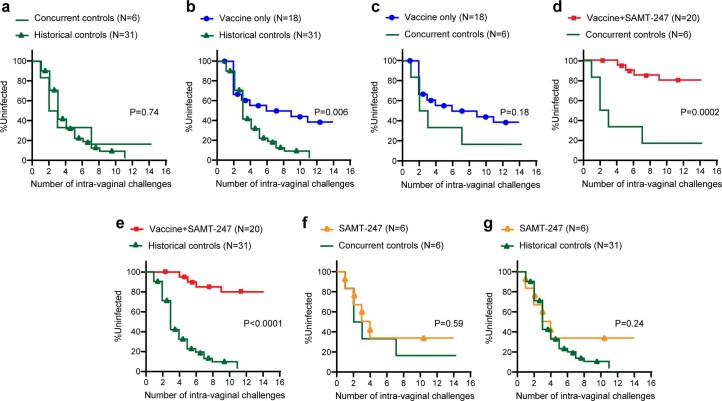
Assessment of infection rate in different animal groups. **a**) No differences in delayed acquisition in the concurrent control group were observed compared to the historical controls (*P* = 0.74). Significant protection or trend of protection in **b, c**) the vaccine group (*P* = 0.006 and *P* = 0.18, respectively) and **d, e**) vaccine+SAMT-247 group (*P* = 0.0002 and *P* < 0.0001, respectively) compared with concurrent or historical controls. **f**, **g**) No differences in delayed acquisition in the SAMT-247 group were observed compared to concurrent (*P* = 0.59) or historical controls (*P* = 0.24). Data shown in (**a-g**) were analyzed with log-rank (Mantel–Cox) test. Source data

We designed a study in macaques powered to dissect the differences between vaccination alone or vaccination plus SAMT-247 treatment, based on the reproducible efficacy of the ΔV1DNA/ALVAC-SIV/ΔV1gp120/alum vaccine in decreasing the risk of SIV_mac251_ acquisition by 60–70% (refs. ^15,16^). We administered the vaccine regimen to 38 female macaques. Five weeks after the last immunization (week 17), all animals were exposed to up to 14 weekly intravaginal SIV_mac251_ challenges. Four hours before each challenge exposure, 20 vaccinated animals were treated vaginally with 0.8% SAMT-247 in hydroxyethyl cellulose (HEC) gel, and the remaining 18 animals with HEC gel only. Two additional groups of non-immunized animals (six each) were treated either with SAMT-247/HEC gel or HEC gel at 4 h before viral exposure as controls (Fig. 1a). All animals were challenged until infection was documented by repeated nanodroplet PCRs. We designed the study to include SIV acquisition data from 31 historical controls (Online Methods) challenged with the same stock of virus in the same animal facility. Vaccine efficacy (VE) was measured as per-exposure risk of SIV acquisition. As expected, no difference in the risk of virus acquisition was observed between concurrent and historical controls (Extended Data Fig. 1a). Vaccine alone significantly (65%) decreased the risk of virus acquisition compared with all controls (*P* = 0.0074; Fig. 1b), as well as only historical controls (*P* = 0.0061; Extended Data Fig. 1b), consistent with prior studies^15^. In addition, a trend was observed using only the six concurrent controls (Extended Data Fig. 1c). Strikingly, the vaccine + SAMT-247 combination afforded a 92.7% reduction in the risk of virus acquisition when compared with all controls (*P* < 0.0001; Fig. 1c), as well as with concurrent or historical controls separately (*P* = 0.0002 and *P* < 0.0001, respectively; Extended Data Fig. 1d,e). The vaccine + SAMT-247 combination differed significantly from the vaccine-only group (*P* = 0.006; Fig. 1d) and protected 16 of 20 animals (80%) from infection. In contrast, treatment with SAMT-247 alone did not significantly decrease the risk of virus acquisition when compared with combined controls (Fig. 1e) or the concurrent or historical controls separately (Extended Data Fig. 1f,g). In vaccinated animals that became infected, we observed lower levels of virus RNA in plasma 2 weeks post-infection, regardless of SAMT-247 treatment, but this was not sustained (Fig. 1f).

### SAMT-247 modulates in vitro NK cell functions

Immune correlates of reduced risk elicited by the DNA/ALVAC/gp120/alum vaccine platform include systemic V2-specific ADCC^16^ mucosal envelope-specific NKp44^+^ cells producing IL-17 (ref. ^13^), CD14^+^ monocytes mediating efferocytosis^15^ and Th1/Th2 cells expressing no or low levels of CCR5 (refs. ^14–16^). We tested the immune correlates of risk separately in vaccinated and vaccinated + SAMT-247-treated groups.

**Extended Data Fig. 2:**
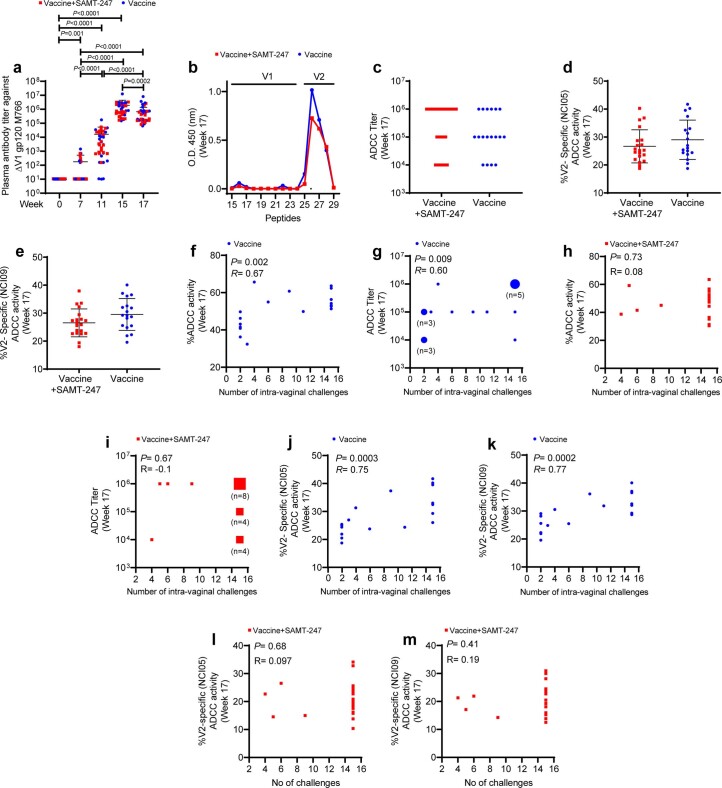
Quantification of humoral responses in plasma of vaccinated rhesus macaques. **a**) Plasma antibody titers against ∆V1 gp120 over the course of immunization in the vaccine+SAMT-247 group (n = 20) and vaccine group (n = 18). **b**) Plasma antibody responses against different peptides encompassing V1 (peptides 15–24) and V2 (peptides 25–29) loop regions of gp120 in the vaccine+SAMT-247 group (n = 20) and vaccine group (n = 18). (**c-e**) Comparison of **c**) ADCC titer (*P* = 0.44), **d**) V2-specific (NCI05-specific) ADCC activity (*P* = 0.33) and **e**) V2-specific (NCI09-specific) ADCC activity (*P* = 0.13) at week 17 in the vaccine+SAMT-247 group (n = 20) and vaccine group (n = 18). (**f-g**) Correlation of **f**) ADCC activity and **g**) ADCC titer in the vaccine group (n = 18). (**h-i**) Correlation of **h**) ADCC activity and **i**) ADCC titer in the vaccine+SAMT-247 group (n = 20), (**j-m**) V2-specific (NCI05 and NCI09-specific) ADCC activity with number of intra-vaginal challenges in the vaccine group (n = 18) and vaccine+SAMT-247 group (n = 20). Data shown in (**a, c-e**) were analyzed with the two-tailed Wilcoxon signed-rank test or two-tailed Mann-Whitney test. Data shown in (**f-m**) were analyzed with the two-tailed Spearman correlation test. Horizontal and vertical bars denote mean and SD. Source data

The levels of systemic antibodies to ΔV1gp120 and V2 peptides, ADCC titres and V2-specific ADCC, measured using a competitive assay with F(ab′)2 obtained from anti-V2 mAbs NCI05 or NCI09, did not differ in the vaccinated SAMT-247-treated or untreated groups, as expected (Extended Data Fig. 2a–e). Surprisingly, specific ADCC activity and titres at the end of immunization (week 17) correlated significantly with decreased risk of SIV_mac251_ acquisition in the vaccine-only group (*R* = 0.67, *P* = 0.002 and *R* = 0.60, *P* = 0.009, respectively; Extended Data Fig. 2f,g), but not in the vaccine+SAMT-247 group (Extended Data Fig. 2h,i). The same was observed for V2-specific ADCC defined by NCI05 and NCI09 F(ab′)2 (*R* = 0.75, *P* = 0.0003 and *R* = 0.77, *P* = 0.0002, respectively; Extended Data Fig. 2j–m). These findings raised the hypothesis that SAMT-247 treatment may have affected mucosal ADCC responses in vivo at the time of virus exposure.

**Fig. 2:**
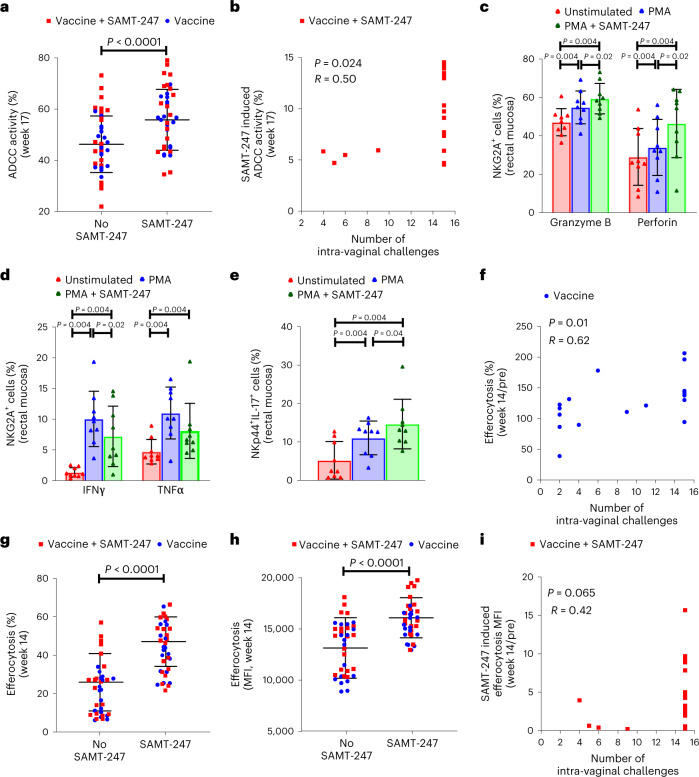
ADCC and NK responses and efferocytosis ex vivo and/or in vitro. **a**, Comparison of SAMT-247 non-treated/treated effector cell-mediated ADCC activity in the vaccine (*n* = 18) and vaccine + SAMT-247 groups (*n* = 20; *P* < 0.0001). **b**, Correlation of SAMT-247-induced ADCC activity with number of intravaginal challenges in the vaccine + SAMT-247 group (*n* = 20; *P* = 0.024). **c**,**d**, Intracellular Granzyme B, perforin, IFN‐γ and TNF-α in macaque rectal mucosal (*n* = 9) NKG2A^+^ cells in the presence or absence of different stimuli. **e**, Macaque rectal mucosal NKp44^+^IL-17^+^ cells in the presence or absence of different stimuli (*n* = 9). **f**, Correlation of efferocytosis with number of intravaginal challenges in animals in the vaccine group (*n* = 18; *P* = 0.01). **g**,**h**, Comparison of percentage of efferocytosis (*P* < 0.0001) (**g**) and efferocytosis MFI (*P* < 0.0001) (**h**) using week 14 CD14^+^ monocytes in all vaccinated animals (*n* = 38). **i**, Correlation of SAMT-247-induced efferocytosis (SAMT-247-untreated efferocytosis subtracted from SAMT-247-treated efferocytosis) with number of intravaginal challenges in the vaccine + SAMT-247 group (*n* = 20; *P* = 0.065). Data shown in **a**, **c**, **d**, **e**, **g** and **h** were analysed with the two-tailed Wilcoxon signed-rank test. Data shown in **b**, **f** and **i** were analysed with the two-tailed Spearman correlation test. Horizontal and vertical bars denote mean and standard deviation, respectively. Source data

**Extended Data Fig. 3:**
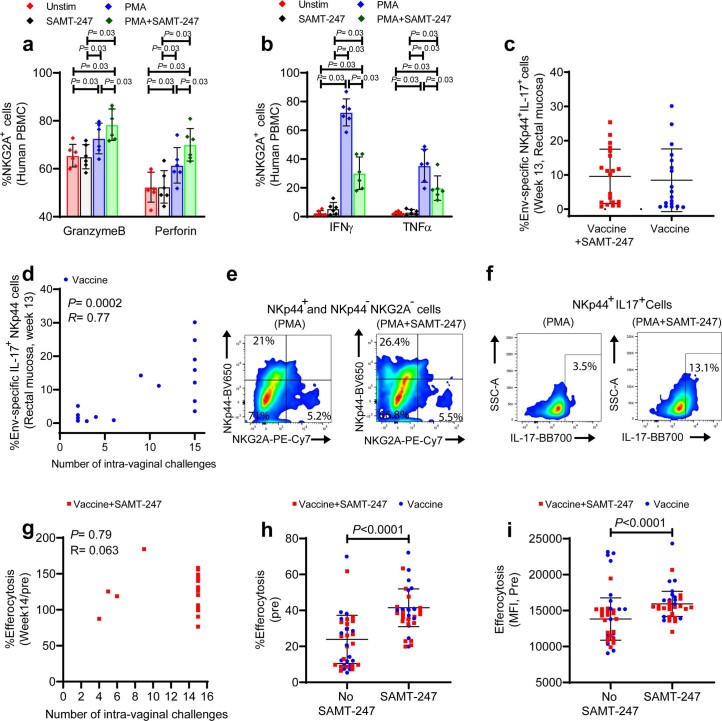
Evaluation of NK/ILCs response and efferocytosis in humans and in rhesus macaques. **a, b)** Intracellular Granzyme B, perforin, IFN‐γ, and TNF-α in healthy human (n = 6) blood NKG2A^+^ cells in the presence or absence of different stimuli. **c**) Comparison of Env-specific rectal NKp44^+^IL-17^+^ cells between vaccine+SAMT-247 (n = 20) and vaccine group (n = 18) 1 week post last vaccination (*P* = 0.43). **d**) Correlation of rectal mucosal Env-specific NKp44^+^IL-17^+^ cells with number of intra-vaginal challenges in the vaccine group (n = 18). **e**) Gating of NKG2A^+^NK cells, NKp44^+^ILCs, and NKG2A^–^NKp44^–^ILCs in rectal mucosal samples in the presence of PMA or PMA + SAMT-247 at 12 hours post stimulation. Gating was done on singlets, live, CD45^+^, CD3^−^, CD20^−^, CD11b^−^ cells. **f**) Gating of NKp44^+^IL-17^+^ ILCs in the rectal mucosal sample in the presence of PMA or PMA + SAMT-247 at 12 hours post stimulation. **g**) Correlation of efferocytosis percentage with number of intra-vaginal challenges in the vaccine+SAMT-247 group (n = 20). (**h-i**) Comparison of **h**) percentage of efferocytosis (*P* < 0.0001) and **i**) efferocytosis MFI (*P* < 0.0001) using pre CD14^+^ monocytes in all vaccinated animals (n = 38). Data shown in (**a, b, h, i**) were analyzed with the two-tailed Wilcoxon signed-rank test or two-tailed Mann-Whitney test. Data shown in (**d, g**) were analyzed with the two-tailed Spearman correlation test. Source data

As mucosal samples from vaccinated animals were not available, we tested this hypothesis by treating human PBMCs with SAMT-247 as effector cells in vitro before setting up the ADCC assays. We found that treatment with SAMT-247 in vitro significantly augmented ADCC activity mediated by plasma antibodies (week 17) from vaccinated animals (*P* < 0.0001; Fig. 2a). The difference in ADCC activity in vitro measured in the presence or absence of SAMT-247 correlated with decreased risk of virus acquisition in vivo in the vaccinated + SAMT-247-treated animals (*R* = 0.50, *P* = 0.024; Fig. 2b). Since these data suggest that topical administration of SAMT-247 may have augmented mucosal natural killer (NK) effector function, we tested whether SAMT-247 also affects mucosal NK function following in vitro PMA (phorbol 12-myristate 13-acetate)/Ionomycin stimulation. For simplicity, PMA/Ionomycin stimulation is referred to as PMA stimulation in this publication. We found that SAMT-247 increased granzyme B and perforin expression (*P* = 0.02 for both; Fig. 2c), and decreased IFN-γ (*P* = 0.02) and TNF-α (not significant (NS)) in macaque mucosal NKG2A^+^ cells (Fig. 2d). A similar effect was also observed in human NK cells from blood (Extended Data Fig. 3a,b). Next, we measured mucosal envelope-specific NKp44^+^IL-17^+^ cells, another correlate of decreased risk of virus acquisition^13,23^, and found that their frequency did not differ in the vaccinated/SAMT-247-treated or untreated groups at week 13, as expected, since at this timepoint no SAMT-247 had been administered (Extended Data Fig. 3c). Interestingly, however, their frequency correlated significantly with decreased risk of virus acquisition in the vaccinated-only group (*R* = 0.77, *P* = 0.0002; Extended Data Fig. 3d), suggesting that this response in the mucosa may have been modulated by SAMT-247 treatment. We tested this hypothesis on mucosal mononuclear cells isolated from nine age-matched macaques by pre-treating them in vitro with PMA with and without SAMT-247. The percentage of NKp44^+^ cells producing IL-17 following PMA stimulation was increased by SAMT-247 (*P* = 0.04; Fig. 2e and Extended Data Fig. 3e,f), supporting the hypothesis that in vivo mucosal treatment with SAMT-247 locally augments the function of NKp44^+^ cells, thereby increasing mucosal integrity and enhancing vaccine efficacy. Collectively, these data suggest that SAMT-247 synergizes with vaccination by increasing NKG2A function and ADCC as well as mucosal protective NKp44^+^ producing IL-17.

### CD14^+^ cell function is increased by SAMT-247

CD14^+^ cell-associated efferocytosis is an innate CD14^+^ monocyte response essential for the clearance of apoptotic cells, maintenance of tissue homeostasis and eradication of pathogens^32^. Vaccine-induced CD14^+^ cell-mediated efferocytosis measured in blood correlated with a reduced risk of virus acquisition in the vaccinated-only group (*R* = 0.62; *P* = 0.01; Fig. 2f), as observed in prior studies with this vaccine regimen^15^, but not in the vaccinated/SAMT-247-treated group (Extended Data Fig. 3g). This raised the hypothesis that SAMT-247 may also affect macrophage functionality at the mucosal site. We therefore performed an efferocytosis assay with the cryopreserved ex vivo CD14^+^ cells purified from blood of vaccinated animals (week 14) in the presence and absence of SAMT-247 and found that SAMT-247 did indeed augment both the percentage of CD14^+^ cells engulfing apoptotic cells as well as the per-cell amount of engulfed apoptotic cells measured as mean fluorescence intensity (MFI) within CD14^+^ cells collected before (pre-)immunization (Extended Data Fig. 3h,i) and at 2 weeks (week 14) following the last immunization (*P* < 0.0001 for both timepoints; Fig. 2g,h). The vaccine-induced capacity of CD14^+^ cells to engulf apoptotic cells following in vitro SAMT-247 treatment trended with a reduced risk of viral acquisition in the animals treated in vivo with SAMT-247 (*R* = 0.42, *P* = 0.065; Fig. 2i).

These data support the hypothesis that SAMT-247 synergizes with vaccine-induced responses at multiple levels: increasing NKG2A^+^ cytotoxic function and V2-specific protective ADCC and enhancing the functionality of protective NKp44^+^ cells and CD14^+^ cells (essential responses to maintain tissue homeostasis and curb inflammation (Extended Data Table 1a).

### SAMT-247 modulates IL-10, IFN-γ and TNF-α expression in CD4^+^ T cells

**Fig. 3:**
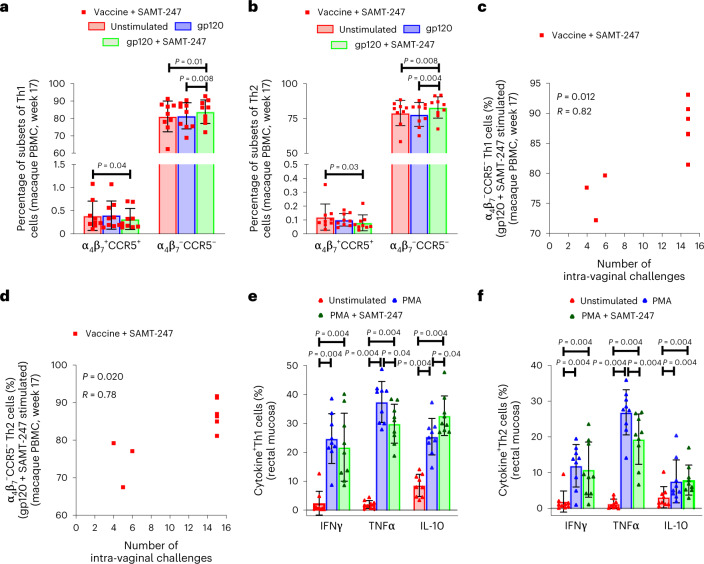
T-cell responses ex vivo and in vitro. **a**,**b**, Evaluation of CCR5 and α_4_β_7_ markers on Th1 and Th2 cells in the absence or presence of stimuli in the vaccine + SAMT-247 group animals (*n* = 9). **c**,**d**, Correlation of gp120 peptide + SAMT-247 stimulated CCR5^−^α_4_β_7_^−^ Th1 (*P* = 0.012) and Th2 cells (*P* = 0.020) with number of intravaginal challenges in the vaccine + SAMT-247 group (*n* = 9). **e**,**f**, IFN‐γ^+^, TNF-α^+^ and IL-10^+^ Th1 and Th2 cells in the rectal mucosa in the absence or presence of stimuli (*n* = 9). Data shown in **a**, **b**, **e** and **f** were analysed with the two-tailed Wilcoxon signed-rank test. Data shown in **c** and **d** were analysed with the two-tailed Spearman correlation test. Horizontal and vertical bars denote mean and standard deviation, respectively. Source data

**Extended Data Fig. 4:**
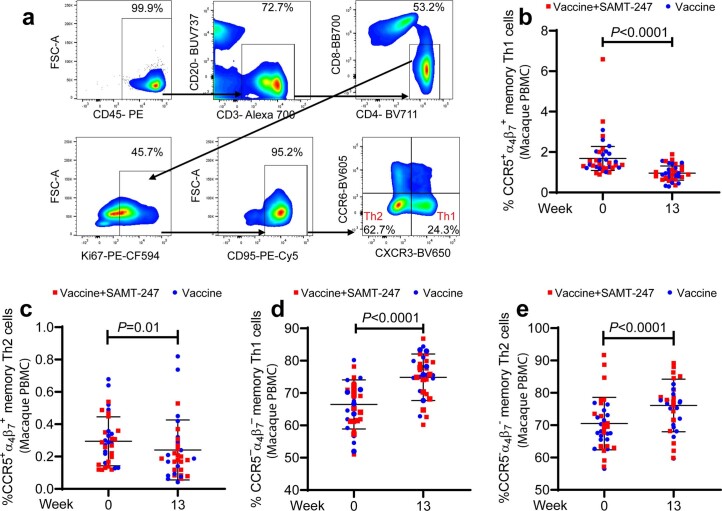
Determination of *ex vivo* macaque T-cell responses. **a**) Gating strategy of Th1 and Th2 cells, **b,c**) Comparison of CCR5^+^α_4_β_7_^+^ (*P* < 0.0001 and *P* = 0.01, respectively) and **d,e**) CCR5^−^α_4_β_7_^-^ memory (*P* < 0.0001 and *P* < 0.0001, respectively) Th1 and Th2 cells pre vaccination and at 1 week post last vaccination (week 13) in blood (n = 38). Data shown in (**b-e**) were analyzed with the two-tailed Wilcoxon signed-rank test. Horizontal and vertical bars denote mean and SD. Source data

**Extended Data Fig. 5:**
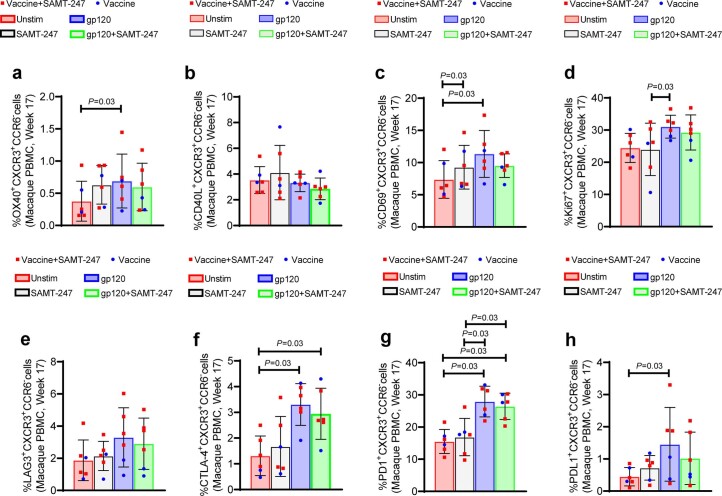
Quantification of activation, proliferation and exhaustion marker on Th1 cells upon stimulation. Expression of **a**) OX40, **b**) CD40L, **c**) CD69, **d**) Ki67, **e**) LAG3, **f**) CTLA-4, **g**) PD-1 and **h**) PDL-1 in Th1 cells upon stimulation in the vaccine+SAMT-247 group (n = 4) and vaccine group (n = 2). Data shown in (**a-h**) were analyzed with the two-tailed Wilcoxon signed-rank test. Horizontal and vertical bars denote mean and SD. Source data

**Extended Data Fig. 6:**
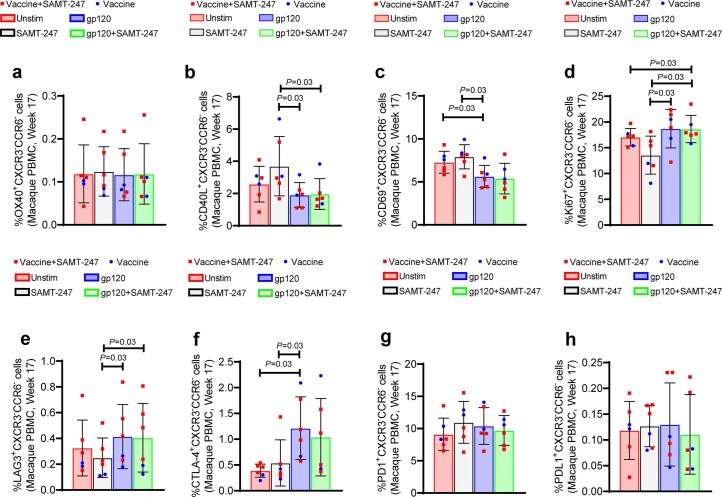
Evaluation of activation, proliferation and exhaustion marker on Th2 cells upon stimulation. Expression of **a**) OX40, **b**) CD40L, **c**) CD69, **d**) Ki67, **e**) LAG3, **f**) CTLA-4, **g**) PD-1 and **h**) PDL-1 in Th2 cells upon stimulation in the vaccine+SAMT-247 group (n = 4) and vaccine group (n = 2). Data shown in (**a-h**) were analyzed with the two-tailed Wilcoxon signed-rank test. Horizontal and vertical bars denote mean and SD. Source data

Vaccine-induced gut homing-activated α_4_β_7_^+^CD4^+^ T cells expressing the SIV/HIV co-receptor CCR5 (α_4_β_7_^+^CCR5^+^ CD4^+^ cells) have been associated with an increased risk of virus acquisition^33,34^. Here we found that vaccination with the ΔV1DNA/ALVAC/ΔV1gp120/alum regimen decreased the frequency of vaccine-induced (Ki67^+^) α_4_β_7_^+^CCR5^+^ memory Th1 (CD4^+^α_4_β_7_^+^CCR5^+^CCR6^−^CXCR3^+^Ki67^+^CD95^+^) and to a lesser extent Th2 (CD4^+^α_4_β_7_^+^CCR5^+^CCR6^−^CXCR3^−^Ki67^+^CD95^+^) cell phenotypes (*P* < 0.0001 and *P* = 0.01, respectively), and significantly increased the frequency of α_4_β_7_^–^CCR5^–^CD4^+^ memory Th1 and Th2 cells in all vaccinated macaques (*P* < 0.0001 for both; Extended Data Fig. 4a–e). To assess the effect of virus acquisition on the activation status of CD4^+^ T cells, we stimulated cryopreserved ex vivo PBMCs from vaccinated animals (week 17) with overlapping peptides encompassing the entire gp120 envelope protein to simulate host response at the time of virus encounter in the presence or absence of SAMT-247. SAMT-247 + gp120 peptides decreased the frequency of vaccine-induced (Ki67^+^) α_4_β_7_^+^CCR5^+^ Th1 and Th2 cells (*P* = 0.04 and *P* = 0.03, respectively; Fig. 3a,b) and increased α_4_β_7_^–^CCR5^–^ Th1 and Th2 cells (*P* = 0.008 and *P* = 0.004, respectively; Fig. 3a,b). Strikingly, at week 17 the percentage of both α_4_β_7_^–^CCR5^–^ Th1 and Th2 cells following in vitro stimulation with gp120 and SAMT-247 significantly correlated with delayed virus acquisition in vivo (*R* = 0.82, P = 0.012 and *R* = 0.76, *P* = 0.020, respectively; Fig. 3c,d). At the same timepoint (week 17), we assessed whether SAMT-247 affected expression of T-cell activation/proliferation/exhaustion on molecules positively and negatively associated with immune responses. This included the receptors/ligand markers OX40, expressed on activated T cells^35^, CD40 ligand (CD154), which triggers a short-term CD4^+^ T-cell activation response^36^, early activation marker CD69 (ref. ^37^), and proliferation marker ki67 (ref. ^38^). We additionally evaluated the negative regulators CTLA-4 and PD-1, which inhibit T-cell activation^39^, the PD-L1/PD-1 pathway, which contributes to T-cell exhaustion^40^, and the exhaustion marker Lag-3 (ref. ^41^). SAMT-247 did not augment gp120 modulation of any one of these molecules (Extended Data Figs. 5 and 6).

**Extended Data Fig. 7:**
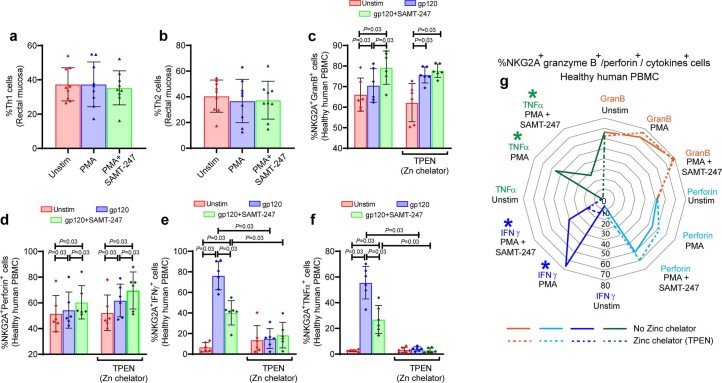
Quantification of macaque mucosal T-cell responses and human NK cell responses. **a, b**) Frequency of Th1 cells and Th2 cells in the rectal mucosa of macaques (n = 9). **c-g**) Comparison of expressions of granzyme B, perforin, IFN‐γ and TNF-α by NKG2A^+^ cells from healthy humans (n = 6) in the absence or presence of zinc chelator and stimuli (n = 6). Data shown in (**a-g**) were analyzed with the two-tailed Wilcoxon signed-rank test. Horizontal and vertical bars denote mean and SD. The radar plot represents the mean percentage value of cytokine responses. Solid lines represent the absence of zinc chelator and dashed lines represent the presence of zinc chelator. Source data

Next, we investigated the effect of SAMT-247 treatment on mucosal Th1 and Th2 cells of age-matched macaques following PMA stimulation. While the overall mucosal Th1 and Th2 cell frequencies did not change upon PMA stimulation (Extended Data Fig. 7a,b), SAMT-247 treatment was associated with a significant decrease in TNF-α production in Th2 cells and more weakly in Th1 cells (*P* = 0.004 and *P* = 0.04, respectively) as well as with increased IL-10 production in Th1 cells (*P* = 0.04; Fig. 3e,f and Extended Data Table 1b).

### Zinc availability and SAMT-247 function

**Fig. 4:**
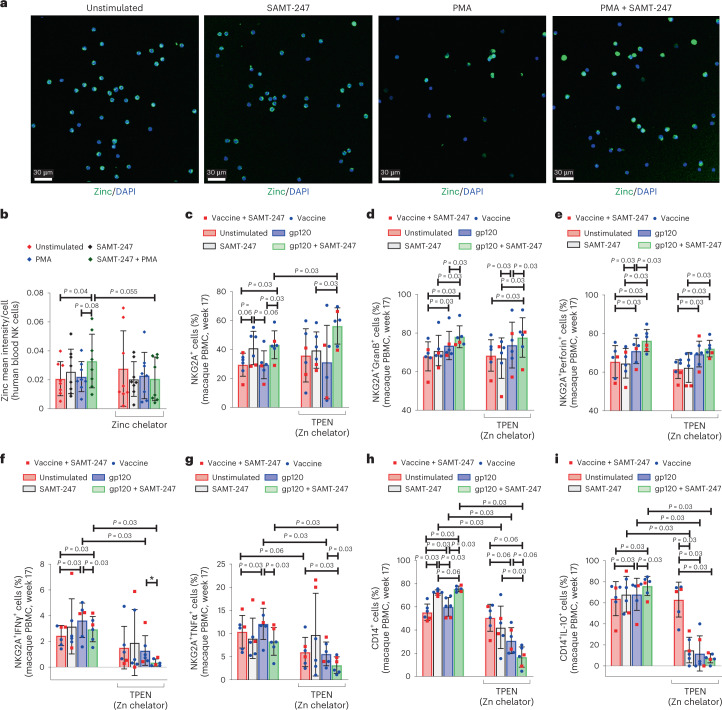
Effect of zinc chelation on NK and monocyte functions. **a**, Representative imaging of human NKG2A^+^ cells unstimulated or stimulated with SAMT-247, PMA or PMA + SAMT-247. **b**, Mean zinc intensity in NKG2A^+^ cells of the healthy human donor in the presence or absence of zinc chelator in different stimulation conditions (*n* = 8). Fluorescence intensity of each field was measured for zinc expression as indicated by green colour, and the total number of DAPI positive cells were counted to determine the mean intensity of zinc/cells using iMARIS software. The mean of two duplicate fields was evaluated for the calculation. **c**, Comparison of expressions of NKG2A marker in macaques in the absence or presence of zinc chelator and stimuli in the vaccine + SAMT-247 group (*n* = 4) and vaccine group (*n* = 2). **d**–**g**, Comparison of expressions of granzyme B, perforin, IFN‐γ and TNF-α by macaque blood NKG2A^+^ cells from week 17 in the absence or presence of different stimulations and zinc chelator in the vaccine + SAMT-247 group (*n* = 4) and vaccine group (*n* = 2). **h**,**i**, Evaluation of the frequency of CD14^+^ monocytes and CD14^+^IL-10^+^ monocytes in the absence or presence of zinc chelator and stimuli in the vaccine + SAMT-247 group (*n* = 4) and vaccine group (*n* = 2). Data shown in **b**–**i** were analysed with the two-tailed Wilcoxon signed-rank test. Horizontal and vertical bars denote mean and standard deviation, respectively. Source data

The spectrum of SAMT-247 effects on NK cell, monocyte and T-cell functions described above suggested that SAMT-247 may affect a central component of immunity. Since zinc is a master regulator of immunity^42,43^, we hypothesized that SAMT-247 may have augmented vaccine-induced immunity by ejecting zinc from proteins and affecting its distribution in immune cells. We used confocal microscopy to measure cellular zinc in human NK cells stimulated with PMA with or without SAMT-247 in the presence or absence of a zinc chelator. PMA + SAMT-247-treated cells had significantly brighter zinc staining per cell compared with unstimulated (*P* = 0.04) and a trend with PMA-stimulated cells (*P* = 0.08; Fig. 4a,b), which trended for a decrease in the presence of the zinc chelator (*P* = 0.055; Fig. 4b).

Next, we investigated the contribution of divalent free zinc to the protective immune responses using cryopreserved ex vivo PBMCs from vaccinated macaques (week 17). To mimic virus exposure, we stimulated cells in vitro with SIV gp120 envelope peptides in the presence or absence of SAMT-247 and of the membrane-permeable intracellular zinc chelator *N*,*N*,*N*′,*N*′ tetrakis-(2-pyridyl-methyl) ethylendiamine (TPEN). Zinc chelation did not affect either SAMT-247-associated NKG2A^+^ survival nor their ability to increase granzyme B and perforin following gp120 peptide stimulation, suggesting that these SAMT-247 activities are not dependent on zinc (Fig. 4c–e). In contrast, TPEN treatment significantly decreased both IFN-γ and TNF-α expression following gp120 peptide or gp120 peptide/SAMT-247 stimulation, suggesting that, by ejecting zinc, SAMT-247 may affect the structural stability of transcription factors for cytokines, and further, that TPEN treatment exacerbates this effect by removing zinc from proteins and sequestering intracellular divalent zinc (Fig. 4f,g). This hypothesis is supported by similar results obtained using human NKG2A^+^ cells (Extended Data Fig. 7c–g).

Several monocyte functions such as monocyte and macrophage phagocytosis are dependent on zinc and can be restored via supplements. Recent studies in humans also demonstrate that the level of intracellular zinc correlates with efferocytosis, itself induced by pro-resolution IL-10 via an IL-10-mediated endocrine mechanism^44–46^. We therefore assessed the effect of zinc chelation on SAMT-247-associated CD14^+^ cell function by stimulating cryopreserved ex vivo CD14^+^ cells from vaccinated animals (pre-immunization and week 13) with gp120 pooled peptides in the presence or absence of SAMT-247 and TPEN. We observed an increased percentage of CD14^+^ cells following SAMT-247 treatment (*P* = 0.03; Fig. 4h), suggesting increased survival of this cell subset in the presence of the drug. However, the frequency of CD14^+^ cells as well as their ability to produce IL-10 greatly diminished following TPEN treatment (Fig. 4i). These data are consistent with prior work demonstrating that divalent zinc increases the survival and functionality of CD14^+^ cells^47^.

**Fig. 5:**
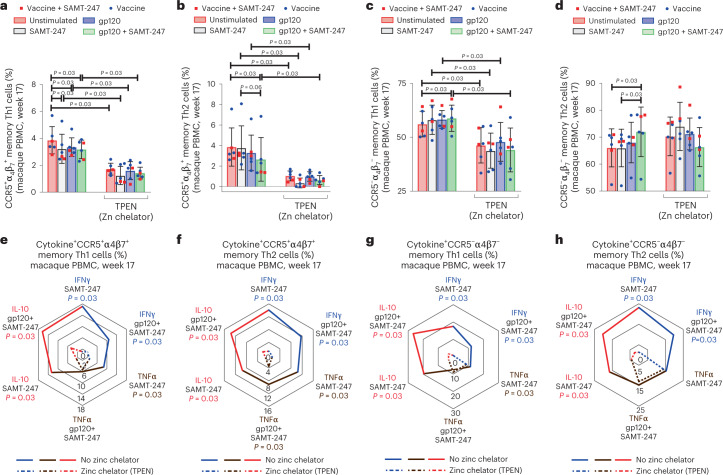
Effect of zinc chelation on CCR5^+/–^ and α_4_β_7_^+/–^ Th1 and Th2 cells. **a**–**d**, Comparison of expressions of CCR5 and α_4_β_7_ markers in Th1 and Th2 memory cells in the absence or presence of zinc chelator and stimuli in the vaccine+SAMT-247 group (*n* = 4) and vaccine group (*n* = 2). **e**–**h**, Radar plots comparing different expressions of cytokines by different subsets of Th1 and Th2 cells from vaccinated animals at week 17 in the absence or presence of stimulation and zinc chelator (*n* = 6). Data shown in **a**–**h** were analysed with the two-tailed Wilcoxon signed-rank test. Horizontal and vertical bars denote mean and standard deviation. The radar plot represents the mean percentage value of cytokine responses. Solid lines represent the absence of zinc chelator and dashed lines represent the presence of zinc chelator. Source data

**Extended Data Fig. 8:**
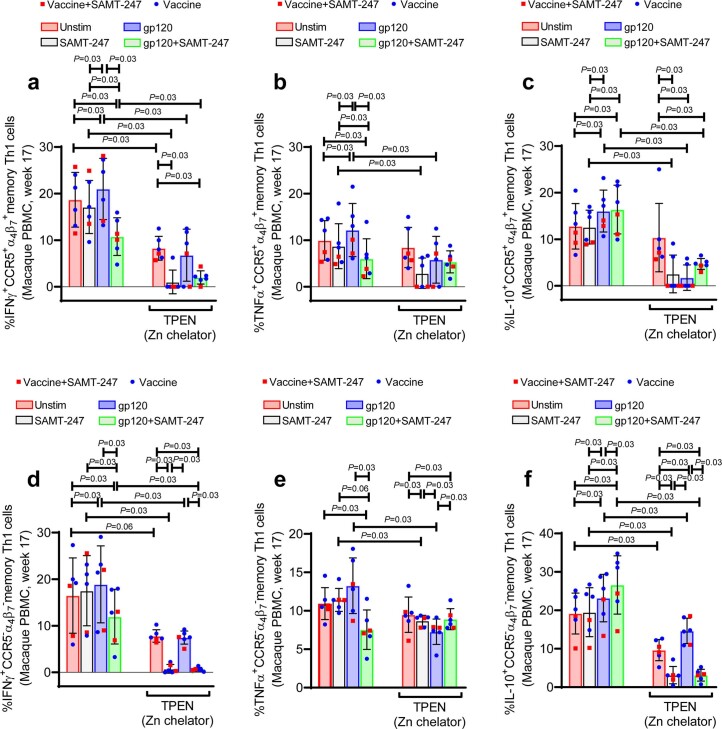
Comparison of cytokine responses by CCR5^+^α_4_β_7_^+^ and CCR5^−^α_4_β_7_^-^ memory Th1 cells in the presence of stimulation. **a-f**) Comparison of intracellular expressions of IFN‐γ, TNF-α, and IL-10 by CCR5^+^α_4_β_7_^+^ and CCR5^−^α_4_β_7_^-^ Th1 memory cells in the absence or presence of zinc chelator and stimuli in the vaccine+SAMT-247 group (n = 4) and vaccine group (n = 2). Data shown in (**a-f**) were analyzed with the two-tailed Wilcoxon signed-rank test. Horizontal and vertical bars denote mean and SD. Source data

**Extended Data Fig. 9:**
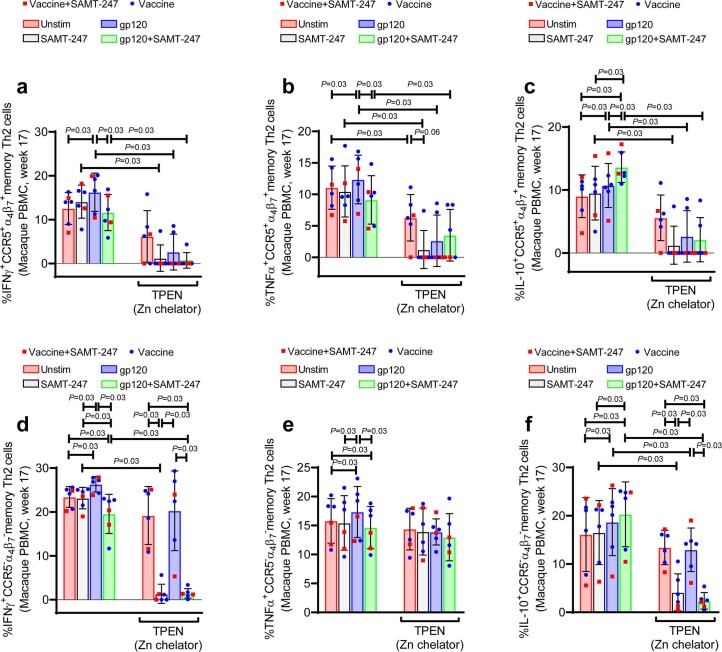
Evaluation of cytokine responses by CCR5^+^α_4_β_7_^+^ and CCR5^−^α_4_β_7_^-^ memory Th2 cells in the presence of stimulation. **a-f**) Comparison of intracellular IFN‐γ, TNF-α, and IL-10 cytokines by CCR5^+^α_4_β_7_^+^ and CCR5^−^α_4_β_7_^-^ Th2 memory cells in the absence or presence of zinc chelator and stimuli in the vaccine+SAMT-247 group (n = 4) and vaccine group (n = 2). Data shown in (**a-f**) were analyzed with the two-tailed Wilcoxon signed-rank test. Horizontal and vertical bars denote mean and SD. Source data

Lastly, we assessed the effect of zinc chelation on cryopreserved ex vivo CCR5 and α_4_β_7_ positive or negative Th1 and Th2 elicited by vaccination (week 17). PBMCs were stimulated with gp120 pooled peptides in the presence or absence of SAMT-247 and TPEN. Zinc chelation resulted in a dramatic decrease of CCR5^+^ α_4_β_7_^+^ CD4^+^ Th1 and Th2 subsets in all conditions (Fig. 5a,b). TPEN caused a significant decrease in the percentage of Th1 negative for CCR5 and α_4_β_7_ expression (*P* = 0.03 for all conditions; Fig. 5c), but did not affect the percentage of Th2 cells negative for CCR5 and α_4_β_7_ expression (Fig. 5d). The expression of IFN-γ, TNF-α and IL-10 was significantly compromised by zinc chelation in Th1 and Th2 cells in most stimulation conditions, regardless of their expression of CCR5 and α_4_β_7_ (Fig. 5e–h and Extended Data Figs. 8 and 9). TNF-α expression in CCR5 and α_4_β_7_ negative Th1 and Th2, however, was not affected with the same severity (Extended Data Figs. 8 and 9).

Collectively, these data demonstrate differential susceptibility of Th1 and Th2 to SAMT-247 and differential dependence on zinc. SAMT-247 in the presence of gp120 peptide increases the viability of Th2 negative for CCR5 and α_4_β_7_ expression and increases expression of IL-10 in all Th1 and Th2 subsets, probably creating an anti-inflammatory environment. IL-10 production is inhibited by zinc chelation; IFN-γ production is highly dependent on zinc in all Th1 and Th2 cell subsets, whereas the decrease in TNF-α expression mediated by SAMT-247 appears to be less zinc dependent (Extended Data Table 1c).

## Discussion

In this pre-clinical macaque study, we investigated whether topical administration of gel-formulated *S*-acyl-2 mercaptobenzamide thioester SAMT-247, which inhibits in vitro HIV maturation and infectivity^48,49^, synergized with DNA/ALVAC/gp120/alum vaccination. As previously reported^15,16^, vaccination alone decreased the risk of SIV_mac251_ acquisition by 65%, and SAMT-247 treatment alone, given 4 h before challenge exposure, had no effect. However, topical SAMT-247 treatment of vaccinated females resulted in a striking 92.7% reduction of per-challenge risk of SIV_mac251_ acquisition, suggesting SAMT-247 additional effects, other than its virucidal activity. We hypothesized that SAMT-247 affected immunity. Using plasma or ex vivo cryopreserved cells from vaccinated animals, respectively, we demonstrated in vitro that SAMT-247 augments NKG2A-mediated ADCC, monocyte efferocytosis and IL-10 production.

Strikingly, SAMT-247 treatment in vitro, also reduced expression of IFN-γ, pro-inflammatory cytokine TNF-α, and CCR5 expression in ex vivo cryopreserved T cells from vaccinated animals, thereby decreasing target cells for SIV infection. Lastly, we demonstrated that in vitro treatment of rectal mucosal cells from macaques increased the frequency of mucosal IL-17-producing NKp44 cells. All of these immune responses have been shown to reproducibly correlate with reduced risk of virus acquisition in vaccinated animals^13–16,23^. Taken together, these findings suggest that SAMT-247’s ability to eject zinc from transcription factors or enzymes may enable zinc-mediated vaccine-induced immunity and protection.

**Fig. 6:**
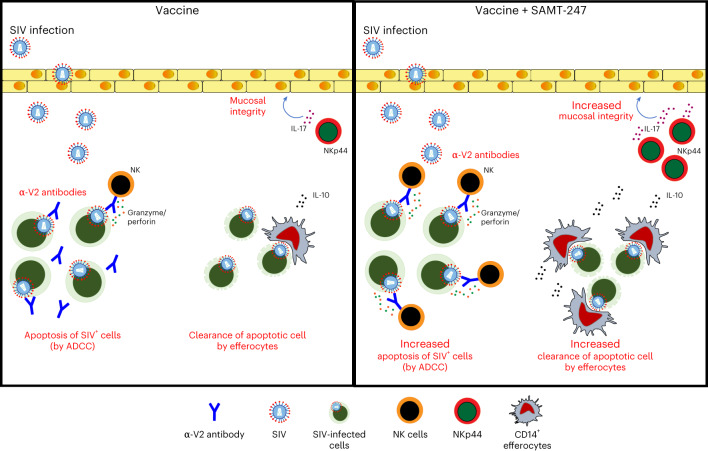
Model for SAMT-247 modulation of mucosal immune responses. Vaccination-induced ADCC results in apoptosis of SIV-infected cells, which in turn are cleared by efferocytes to avoid inflammation and preserve tissue homeostasis. Vaccine-induced IL-10 expression in CD14^+^ monocytes further augments efferocytosis. Vaccine-induced NKp44^+^ cells produce the IL-17 cytokine that maintains mucosal epithelium integrity. All of these protective effector responses were enhanced dramatically in the vaccine + SAMT-247 group, increasing protection from SIV_mac251_ acquisition. The scheme is adapted from Bissa et al.^15^.

Zinc is an essential micronutrient and is a structural constituent in approximately 800 zinc-finger transcription factors^50^ and 2,000 enzymes^51^. Its role in immunity is well known^42,43,52,53^. Relevant to the vaccine approach tested here, zinc and zinc transporters have a role in efferocytosis^46^ and zinc supplementation augments granulocyte and monocyte phagocytic function^54^. Intracellular zinc mobilization is triggered by the activation of the c-AMP pathway in human pathogens^55^, and a dysfunction of Th2 responses linked to defective reprogramming of monocytes to anti-inflammatory M2 macrophages has been demonstrated in mice fed a zinc-deficient diet^56^. Indeed, we found that in vitro chelation of zinc by TPEN affected SAMT-247 modulation of IFN-γ, TNF-α and CCR5 expression in stimulated T cells, as well as IL-10 in monocytes. In contrast, zinc chelation did not affect the SAMT-247-associated increase of perforin and granzyme in NK cells (Extended Data Table 1c). Therefore, we conclude that SAMT-247 synergizes with the DNA/ALVAC/gp120/alum vaccine regimen as an immune enhancer by augmenting the immunological function of effector cells (Fig. 6).

We cannot however exclude a possible contribution of the anti-viral effect of SAMT-247 in reducing virus infectivity in the vaginal mucosa. In our study, we did not observe an effect on virus acquisition by SAMT-247 alone in naïve animals, though it must be noted that the protective effect of SAMT-247 alone previously reported in the SIV_mac251_ model was obtained in animals mock-immunized with empty Ad vector and, relevant to the alum adjuvant^31^, a key contributor to the immunological landscape created by the DNA/ALVAC/gp120/alum vaccine.

In summary, we provide evidence that a combination of the V1-deleted envelope virus-like particle delivered with the DNA/ALVAC/gp120/alum vaccine platform, and SAMT-247 is highly efficacious in preventing vaginal infection by a neutralization-resistant, highly pathogenic virus. Our data also suggest that ADCC and efferocytosis may be true effectors of protection against SIV/HIV, pointing to an underestimated role of monocytes and NK cells in vaccine efficacy. Finally, our data underscore the importance of pro-resolution anti-inflammatory responses able to maintain low levels of T-cell activation in protection from SIV/HIV acquisition.

## Methods

This research complies with all relevant ethical regulations. The NCI Animal Care and Use Committee (ACUC) approved the vaccine study. Nine additional animal tissues were obtained from our collaborative study with the Tulane animal facility, and the study was approved by the Tulane ACUC. Human PBMCs were obtained from eight healthy donors via an institutional review board-approved National Institutes of Health (NIH) protocol.

### Animals

Fifty female Indian rhesus macaques obtained from the free-range breeding colony on Morgan Island, South Carolina, were used in this study. The macaques, aged 2–3 years at study initiation, were negative for SIV, simian retrovirus and Simian T-Cell Leukemia Virus (STLV), and were MHC typed for Mamu A*01, Mamu B*17 and Mamu B*08. The macaques were randomized by the statistician on the basis of age, weight and haplotype before being divided into four groups: vaccine/microbicide group (20 macaques, 3 A*01 positive, 1 B*17 positive and 1 B*08 positive); vaccine-only group (18 macaques, 3 A*01 positive and 1 B*17 positive); microbicide-only group (6 macaques, 1 both A*01 positive and B*17 positive) and control group (6 macaques, 1 both A*01 positive and B*17 positive). These group sizes were determined on the basis of a previous vaccine study comparing 14 vaccinated macaques and 18 controls^15,16^, and on a pilot experiment in which we defined a time between microbicide administration and viral challenge that would decrease the microbicide efficacy so that we could observe an additive/synergistic effect^31^. We selected a 4 h ‘window’ between administration of microbicide and viral challenge, as this time frame was demonstrated ineffective with SAMT-247 alone^31^. The historical control macaques and microbicide macaques were also used in the statistical analysis. Infection rates were assumed to equal those of the previously observed gel-only and microbicide groups, 0.333 and 0.133, respectively, and the vaccine and combination groups, 0.120 and 0.040. The treatment efficacies of the latter three groups relative to the gel-only group were expected to be 60% (microbicide), 64% (vaccine) and 88% (combination). When the animals were given up to 14 viral challenges and the number of challenges to infect the animals was compared using the Wald test of the proportional hazards model with all four groups analysed together, then the power of the test at the two-sided 0.05 level between the vaccine group and the combination group was expected to be 79%, while the comparisons of these two groups individually versus the control group would respectively have 82% and 99% power. The test of the microbicide group versus the combination group would have 63% power. Data on 31 historical naïve controls intravaginally challenged with the same dose and lot of SIV_mac251_ were added to the control group to increase statistical power. There was no difference between the concurrent and historical controls in rate of SIV acquisition (*P* = 0.74). Macaques were housed and maintained at the NCI Animal Facility at the NIH, Bethesda, MD. All animals were handled in accordance with the standards of the Association for the Assessment and Accreditation of Laboratory Animal Care (AAALAC) in an AAALAC-accredited facility (OLAW, Animal Welfare Assurance A4149-01 for NIH). All animal care and procedures were carried out under protocols approved by the NCI ACUC before study initiation. Animals were closely monitored daily for any signs of illness, and appropriate medical care was provided as needed. Animals were socially housed per the approved ACUC protocol and social compatibility except during the viral challenge phase when they were individually housed. All clinical procedures, including biopsy collection, administration of anaesthetics and analgesics, and euthanasia, were carried out under the direction of a laboratory animal veterinarian. Steps were taken to ensure the welfare of the animals and minimize discomfort of all animals used in this study. Animals were fed daily with a fresh diet of primate biscuits, fruit, peanuts and other food items to maintain body weight or normal growth. Animals were monitored for psychological wellbeing and provided with physical enrichment including sanitized toys, destructible enrichment (cardboard and other paper products) and audio and visual stimulation.

Additionally, rectal tissue from nine random female rhesus macaques aged 2–3 years was used for an in vitro study. The age-matched macaques from this separate study included four animals vaccinated against HIV and five naïve animals, all challenged with Simian-human immunodeficiency viruses (SHIV). Rectal tissue was obtained post challenge from seven infected and two uninfected animals for the analysis of this study. Extra cells were available from that collection and were used here for the in vitro study. Macaques were housed and maintained at the Tulane University animal facility. The nine animals were handled in accordance with the standards of the Association for the Assessment and Accreditation of Laboratory Animal Care in an AAALAC-accredited facility (OLAW, Animal Welfare Assurance). The care of the nine animals and all procedures were carried out under protocols approved by the Tulane ACUC.

### Human healthy volunteers

Human PBMCs were obtained from eight healthy donors on an institutional review board-approved NIH protocol (99-CC-0168). Research blood donors provided written informed consent and blood samples were de-identified before distribution. Clinical Trial Number: NCT00001846.

### Immunization and challenge

Macaques in the vaccine/microbicide and vaccine-only groups were immunized at weeks 0 and 4 with DNA encoding SIVgp160ΔV1 (2 mg per dose) and SIV239gag (1 mg per dose) in a total volume of 1 ml PBS. The DNA was administered in both thighs (0.5 ml to each). At 8 weeks the macaques were administered ALVAC encoding *gag/pro/env* (wild-type *env*) in the right thigh, 10^8^ pfu per dose in 1 ml PBS. At week 12 the macaques were boosted with the same ALVAC plus SIVgp120ΔV1 protein (400 µg per dose in 500 μl PBS plus 500 μl 2% Alhydrogel). The ALVAC was administered to the right thigh; the 1 ml dose of Env protein plus alum was administered to the left thigh. Beginning at week 17 all macaques were challenged intravaginally weekly with 1 ml of a SIV_mac251_ stock containing 4000 TCID_50_/mL (evaluated in rhesus 221 cells). Up to 14 challenges were administered until the macaques became SIV positive as assessed by droplet digital PCR (H. K. Chung, J. Narola, H. Babbar, M. Naseri, N. Richardson, R. Pal and T. Fouts, manuscript in preparation). Doses of 2 ml of the microbicide, SAMT-247, were administered as a gel intravaginally to macaques in the vaccine/microbicide group and the microbicide-only group 4 h before each SIV challenge. The 2 ml gel contained 0.8% SAMT-247 in HEC gel (2.7% Natrosol cellulose 250HX Pharma, 0.01% DMSO and 0.9% saline). Macaques in the vaccine-only group and control group received HEC gel lacking only the SAMT-247. Rhesus macaques were vaccinated and samples were collected during the vaccination period as well as following vaginal exposure of the animals to SIV_mac251_. The same material cannot be obtained multiple times from the same monkey, making these biological specimens unique.

### IgG plasma titres to gp120

gp120 total IgG antibodies were measured by ELISA. ELISA plates (Nunc Maxisorp 96-well plate) were coated with 100 μl of 500 ng ml^−1^ SIV_mac251-M766_ gp120 protein per well in 50 mM sodium bicarbonate buffer pH 9.6 and incubated overnight at 4 °C. Plates were blocked with 200 μl PBS Superblock (Thermo Fisher Scientific) for 1 h at room temperature. Plasma samples were serial-diluted with sample diluent (Avioq), and 100 μl of diluted plasma was added to the wells. Plates were covered and incubated for 1 h at 37 °C, washed six times with PBS Tween 20 (0.05%) and incubated with 100 μl of anti-human HRP diluted at 1:120,000 in sample diluent (Avioq) for 1 h covered at 37 °C. The plates were washed six times. Plates were developed using 100 μl K-Blue Aqueous substrate (Neogen) to all wells and incubated for 30 min at room temperature. The reaction was stopped by the addition of 100 μl of 2 N sulfuric acid to all wells and the plate was read at 450 nm on a Molecular Devices E-max plate reader.

### Pepscan

Plasma samples were assayed by PEPSCAN analysis using SIV_mac251_ gp120 linear peptides^16^. ELISA plates (Nunc Maxisorp) were coated with 100 ng of each of the 1–89 overlapping peptides (with 15 amino acids each encompassing the entire SIV_mac251_ gp120 sequence) in 50 mM NaHCO3, pH 9.6, per well, incubated overnight at 4 °C, and blocked with 200 μl of Pierce SuperBlock blocking buffer in PBS for 1 h at room temperature. Serum samples were diluted at 1:50 in sample diluent (Avioq), and 100 μl were added to the plate and incubated for 1 h at 37 °C. Plates were washed six times with PBS Tween 20 (0.05%) and incubated with 100 μl anti-human HRP diluted at 1:120,000 in sample diluent (Avioq) to all wells and incubated, covered, for 1 h at 37 °C. The plates were again washed six times and developed using 100 μl of K-Blue Aqueous substrate (Neogen) to all wells and incubated 30 min at room temperature. The reaction was stopped by adding 100 μl of 2 N sulfuric acid to all wells and plate was read at 450 nm on a Molecular Devices E-max plate reader.

### Rectal mucosal NK/ILC phenotyping and cytokine expression upon gp120 peptides/PMA-Ionomycin stimulation in vaccinated animals

The frequency and cytokine levels of NK/ILCs were measured in macaque rectal mucosa pre-vaccination and 1 week post last vaccination (week 13). Freshly collected rectal biopsies were treated with collagenase (2 mg ml^−1^; Sigma-Aldrich) in the absence of FBS in 37 °C for 1 h, then mechanically separated by using a 10 ml syringe with a blunt head canula. Biopsies were washed with R10 and passed through a 70 μm cell strainer. Single cells were counted and used for the experiment^23^. A portion of the cells were phenotyped and the rest were cultured in R10 in the presence/or absence of gp120 peptides or PMA (phorbol 12-myristate 13-acetate)-Ionomycin for 12 h. Subsequently, cells were stained with Live/Dead Aqua Dye (cat. no. L34966, 0.5 μl) from Thermo Fisher, followed by surface staining with the following: Alexa 700 anti-CD3 (SP34-2; cat. no. 557917, 5 μl), Alexa 700 anti-CD20 (2H7; cat. no. 560631, 5 μl), Alexa 700 anti-CD11b (ICRF44; cat. no. 557918, 5 μl), APC-Cy7 anti-CD16 (3G8; cat. no. 557758, 5 μl), PE-CF594 anti-CD56 (B159; cat. no. 562289, 5 μl) BV650 anti-NKp44 (P44-8; cat. no. 744302, 5 μl), BV786 anti-CD45 (D058-1283; cat. no. 563861, 5 μl) from BD Biosciences; and PE-Cy7 anti-NKG2A (Z199; cat. no. B10246, 5 μl) from Beckman Coulter for 30 min at room temperature. This was followed by permeabilization with a FOXP3-transcription buffer set (cat. no. 00−5523-00) from eBioscience according to the manufacturer’s recommendation and subsequently intracellular staining with the following: BV421 anti-IFN-γ (B27; cat. no. 562988, 5 μl) from BD Biosciences and PE-Cy5.5 anti-IL-17 (BL168; cat. no. 512314, 5 μl) from BioLegend for 30 min at room temperature. Samples were acquired on a BD FACSymphony A5 cytometer and analysed with FlowJo software 10.6. NKG2A^+^ NK cells were gated as singlets, live cells, CD45^+^ cells, CD3^−^, CD20^−^, CD11b^−^ and NKG2A^+^ NKp44^−^ cells. NKp44^+^ cells were gated as singlets, live cells, CD45^+^ cells, CD3^−^, CD20^−^, CD11b^−^ and NKG2A^−^ NKp44^+^ cells. NKG2A^−^ NKp44^−^ cells were gated as singlets, live cells, CD45^+^ cells, CD3^−^, CD20^−^, CD11b^−^ and NKG2A^−^ NKp44^−^ cells. Cytokines were gated on parent population.

### CD4^+^ T-cell phenotypes

The levels of CD4^+^ T-cell subsets were measured in blood at baseline and week 13 in vaccinated animals. PBMCs were stained with the following: LIVE/DEAD Fixable Blue Dead Cell Stain (cat. no. L23105, Thermo Fisher); Alexa 700 anti-CD3 (SP34-2; cat. no. 557917, 5 μl), BV785 anti-CD4 (L200; cat. no. 563914, 5 μl), PeCy5 anti-CD95 (DX2; cat. no. 559773, 5 μl), BV650 anti-CCR5 (3A9; cat. no. 564999, 5 μl), BUV496 anti-CD8 (RPA-T8; cat. no. 564804, 5 μl), BUV737 anti-CD28 (CD28.2; cat. no. 612815, 5 μl) and FITC anti-Ki67 (B56; cat. no. 556026, 5 μl) from BD Biosciences; APC Cy7 anti-CXCR3 (G025H7; cat. no. 353722, 5 μl), BV605 anti-CCR6 (G034E3; cat. no. 353420, 5 μl), BV510 anti-CD127 (A019D5; cat. no. 351332, 5 μl), BV750 anti-PD-1 (EH12.2H7; cat. no. 329965, 5 μl) and BV711 anti-CD25 (BC96; cat. no. 302636, 5 μl) from BioLegend; PE-eFluor 610 anti-CXCR5 (MU5UBEE; cat. no. 61-9185-42, 5 μl), eFluor 450 anti-FoxP3 (236A/E7; cat. no. 48-4777-42, 5 μl) from eBioscience; and APC anti-α_4_β_7_, provided by the NIH Nonhuman Primate Reagent Resource (R24 OD010976, and NIAID contract HHSN272201300031C). Samples were acquired on a BD FACSymphony A5 cytometer and analysed with FlowJo software 10.6. Gating was done on live CD3^+^CD4^+^ cells and on vaccine induced Ki67^+^ cells. CXCR3 and CCR6 expression were used to identify Th1 or Th2 populations^14^.

### ADCC CEM-based assay

ADCC activity was assessed using EGFP-CEM-NKr-CCR5-SNAP cells that constitutively express GFP as targets^23,57^. Briefly, one million target cells were incubated with 50 μg of ΔV1 gp120 protein for 2 h at 37 °C. After this coating, the target cells were washed and labelled with SNAP-Surface Alexa Fluor 647 (New England Biolabs) per manufacturer recommendations for 30 min at room temperature. Plasma samples, heat inactivated at 56 °C for 30 min, were serially diluted (seven ten-fold dilutions starting at 1:10) and 100 μl were added to wells of a 96-well V-bottom plate (Millipore Sigma). A total of 5,000 target cells (50 μl) and 250,000 human PBMCs (50 μl) were added as effectors to each well to give an effector/target (E/T) ratio of 50:1. The plate was incubated at 37 °C for 2 h followed by two PBS washes. The cells were resuspended in 200 μl of a 2% PBS–paraformaldehyde solution and acquired on a Symphony equipped with a high-throughput system (BD Biosciences). Specific ADCC activity was measured by loss of GFP from the SNAP-Surface Alexa647^+^ target cells. Target and effector cells cultured in the presence of R10 medium were used as background. Anti-SIVmac gp120 monoclonal antibody KK17 (NIH AIDS Reagent Program) was used as a positive control. Normalized ADCC activity was calculated as: (ADCC activity in the presence of plasma − background)/(ADCC activity in the presence of KK17 − background) × 100. The normalization was done to minimize plate-to-plate and experiment-to-experiment variation of the assay. The ADCC endpoint titre is defined as the reciprocal dilution at which the percent ADCC activity was greater than the mean percent ADCC activity of the background wells containing medium only with target and effector cells, plus three standard deviations.

### Inhibition of ADCC CEM-based assay by monoclonal F(ab′)2 of NCI05 and NCI09

F(ab′)2 fragments were prepared from both NCI05 or NCI09 mAb, as these antibodies recognize overlapping conformationally distinct V2 epitopes^16^, using Pierce F(ab′)2 Micro Preparation Kit (cat. no. 44688, Thermo Fisher) following the manufacturer’s instructions. An SDS–PAGE gel with the recovered F(ab′)2 was run and Silver stained (cat. no. LC6070, Silver Quest staining Kit, Invitrogen) according to the manufacturer’s instructions, to assure the purity of the F(ab′)2 fragments. Target cells, coated with ΔV1 gp120 protein as indicated above and labelled with SNAP-Surface Alexa Fluor 647, were incubated for 1 h at 37 °C with 5 μg ml^−1^ of purified F(ab′)2 fragments from NCI05, or NCI09 monoclonal antibodies. Cells incubated without F(ab′)2 served as control. These target cells were subsequently used in the ADCC assay as described above. These F(ab′)2 inhibit binding (and ADCC) mediated by the anti-V2 antibodies from immunized animals’ plasma. The percentage ADCC activity difference in the presence or absence of F(ab′)2 is considered V2-specific ADCC activity.

### ADCC assay using SAMT-247

Ten million human PBMC effector cells were resuspended in 1 ml of R10 medium and incubated in the presence/absence of 100 μM of SAMT-247 for 4 h. Cells were washed and used as effector cells to measure ADCC activity as described above. SAMT-247-induced ADCC was measured by subtracting ADCC without SAMT-247-treated effector cells from ADCC with SAMT-247-treated effector cells.

### Efferocytosis assay

The frequency of efferocytotic CD14^+^ cells was assessed by Efferocytosis Assay kit (cat. no. 601770, Cayman Chemical Company). CD14^+^ cells were used as effector cells, whereas apoptotic neutrophils were used as target cells. The protocol was re-adapted to use CD14^+^ monocyte cells rather than differentiated macrophages due to the low cell availability^15^. CD14^+^ cells were isolated from cryopreserved PBMCs (10 × 10^6^ cells) collected following pre-study and 2 weeks post last immunization (week 14) by using non-human primate CD14 MicroBeads (#130-091-097, Miltenyi Biotec) following manufacturer instructions. At the end of the separation, cells were counted and stained with CytoTell Blue (AAT Bioquest) provided by the kit and following manufacturer instructions. One unrelated macaque was used as source of neutrophils as target cells. Neutrophils were isolated of PBMCs by Ficoll Plaque (GE Healthcare), the cellular pellet was added to an equal volume of 20% dextran in water, gently mixed, and incubated for 1 min. Approximately three volumes of PBS were added, mixed again, and incubated in the dark for 50–60 min. At the end of incubation, the clear layer at the top of the tube containing neutrophils was collected. Cells were pelleted and treated with ACK lysing buffer (Quality Biological) for 5 min at 37 °C, washed with R10 and counted. Neutrophils were stained with CFSE provided by the kit following manufacturer instructions. The apoptosis of neutrophils was induced by treatment with Staurosporine Apoptosis inducer provided by the kit. Briefly, isolated cells were resuspended in R10 containing Staurosporine diluted 1:1,000 and incubated at 37 °C for 3 h. At the end of the incubation cells were washed twice with R10 and used for the efferocytosis assay. Subsequently, effector and apoptotic target cells were cultured alone (as controls) or co-cultured at a ratio of one effector CD14^+^ cell to three target apoptotic neutrophils. Cells were incubated at 37 °C for 12 h. At the end of the co-culture, cells were washed with PBS, fixed with 1% paraformaldehyde in PBS and acquired on a FACSymphony A5 and examined using FACSDiva software (BD Biosciences) by acquiring all stained cells. Data were further analysed using FlowJo v10.6 (TreeStar). The frequency of efferocytotic CD14^+^ cells was determined as the frequency of double-positive cells for CytoTell Blue and CFSE on the CytoTell Blue-positive monocytes. The efferocytosis assay was done in several experiments, each time paired pre- and post-vaccination was done together. Thus, to combine the experiments for correlation analysis, the post-vaccination efferocytosis (week 14) data were normalized by dividing them with pre-vaccination data and multiplied by 100.

### Efferocytosis assay using SAMT-247

Efferocytosis was done as described above with the exception that 100 μM of SAMT-247 were added to the co-culture for 12 h. SAMT-247-induced efferocytosis was measured by subtracting efferocytosis without SAMT-247-treated effector cells from efferocytosis with SAMT-247-treated effector cells.

### Intracellular cytokines of human blood NK cells using SAMT-247 and PMA-Ionomycin stimulation with or without zinc chelator

The levels of NKG2A^+^ NK cells were measured in blood of healthy humans. Human PBMCs were thawed and cultured in R10 in the presence or absence of SAMT-247 and/or PMA-Ionomycin for 12 h. Cells were incubated with or without zinc chelator TPEN (cat. no. P4413-100MG, 5 μM) for 12 h. Subsequently, PBMCs were surface stained with the following: BUV737 anti-CD3 (SP34-2; cat. no. 741872, 5 μl), Alexa 700 anti-CD20 (2H7; cat. no. 560631, 5 μl), BV786 anti-CD45 (HI30; cat. no. 563716, 5 μl) from BD Biosciences; APC-H7 anti-CD11b (ICRF44; cat. no. 47-0118-42, 5 μl) from eBioscience and PE-Cy7 anti-NKG2A (Z199; cat. no. B10246, 5 μl) from Beckman Coulter for 30 min at room temperature. This was followed by permeabilization with FOXP3-transcription buffer set (cat. no. 00-5523-00) from eBioscience according to the manufacturer’s recommendation and subsequent intracellular staining with the following: BV750 anti-TNF-α (MAB11; cat. no. 566359, 5 μl), BUV396 anti-IFN-γ (B27; cat. no. 563563, 5 μl), BV510 anti-GranB (GB11; cat. no. 563388, 5 μl) from BD Biosciences; and FITC anti-Perforin (pf-344; cat. no. 3465-7, 5 μl) from MABTECH for 30 min at room temperature. Flow cytometry acquisitions were performed on a FACSymphony A5 and examined using FACSDiva software (BD Biosciences).

### AIM assay

Activation, proliferation and exhaustion markers on Th1 and Th2 cell subsets were measured in blood at week 17 in vaccinated animals using AIM assay^58^. PBMCs were thawed and incubated with CD40 blocking antibody (HB14, cat. no. 130-094-133, 5 μl) from Miltenyi for 15 min, followed by the addition of CD49a (9F10, cat. no. 555501, 2 μl) and CD28 (CD28.2, cat. no. 567117, 2 μl) from BD Bioscience in the presence or absence of gp120 and/or SAMT-247. Cells were incubated at 37 °C for 18 h followed by antibody staining. PBMCs were stained for 30 min at room temperature with the following: LIVE/DEAD Fixable Blue Dead Cell Stain (cat. no. L23105, Thermo Fisher); BV786 anti-CD45 (D058-1283; cat. no. 563861, 5 μl), BUV737 anti-CD3 (SP34-2; cat. no. 741872, 5 μl), BV711 anti-CD4 (L200; cat. no. 740807, 5 μl), BUV496 anti-CD8 (RPA-T8; cat. no. 612942, 5 μl), PE-CF594 anti-PDL1 (MIH1; cat. no. 563742, 5 μl), BB700 anti-CTLA-4 (BNI3; cat. no. 566901, 5 μl), PE-Cy5 anti-OX40 (CD134) (ACT35; cat. no. 551500, 5 μl), BUV563 anti-CD40L (CD154) (24-31; cat. no. 752854, 5 μl), PE-Cy7 anti-CD69 (FN50; cat. no. 557745, 5 μl), PE anti-CD95 (DX2; cat. no. 555674, 5 μl) from BD Bioscience; FITC anti-LAG3 (3DS223H; cat. no. 369326, 5 μl) from Thermofisher; BV750 anti-PD1 (EH12.2H7; cat. no. 329966, 5 μl), Alexa 700 anti-CXCR3 (G025H7; cat. no. 353742, 5 μl), BV605 anti-CCR6 (G034E3; cat. no. 353420, 5 μl) from BioLegend. Staining was followed by permeabilization with a FOXP3-transcription buffer set (cat. no. 00-5523-00) from eBioscience according to the manufacturer’s recommendation and subsequently stained intracellularly with BV510 anti-Ki67 (B56; cat. no. 563462, 5 μl) from BD Biosciences for 30 min at room temperature. Samples were acquired on a BD FACSymphony A5 cytometer and analysed with FlowJo software 10.6. Th1 cells were gated as singlets, live cells, CD45^+^ cells, CD3^+^, CD4^+^, CD8^−^, CD95^+^, CXCR3^+^, CCR6^−^; Th2 cells were gated as singlets, live cells, CD45^+^ cells, CD3^+^, CD4^+^, CD8^−^, CD95^+^, CXCR3^−^, CCR6^−^. Activation, proliferation and exhaustion markers were gated on parent population.

### Intracellular cytokines of macaque blood NK cells and T cells using SAMT-247 and gp120 stimulation with or without zinc chelator

The levels of NKG2A^+^ NK cells and T cells were measured in blood of vaccinated macaques. Macaque PBMCs were thawed and cultured in R10 in the presence or absence of SAMT-247 and/or gp120 pooled peptides for 12 h. Cells were incubated with or without zinc chelator TPEN (cat. no. P4413-100MG, 5 μM) for 12 h. Subsequently, cells were stained for live cells with Live/Dead Blue dye (cat. no. L34962, 0.5 μl) from Thermo Fisher; followed by surface staining with the following: PE anti-CD45 (D058-1283; cat. no. 552833, 5 μl), BB700 anti-CD3 (RPA-T8; cat. no. 566452, 5 μl), Alexa 700 anti-CD3 (SP34-2; cat. no. 557917, 5 μl), BV711 anti-CD4 (L200; cat. no. 563913, 5 μl), BV786 anti-CCR5 (3A9; cat. no. 565001, 5 μl), BUV737 anti-CD20 (2H7; cat. no. 612848, 5 μl), BUV496 anti-CD16 (3G8; cat. no. 612944, 5 μl), BUV661 anti-HLA-DR (G46-6; cat. no. 612980, 5 μl), BUV805 anti-CD14 (M5E2; cat. no. 565779, 5 μl) from BD Biosciences; APC-H7 anti-CD11b (ICRF44; cat. no. 47-0118-42, 5 μl), PE-Cy5 anti-CD95 (DX2; cat. no. 15-0959-42, 5 μl) from eBioscience; PE-Cy7 anti-NKG2A (Z199; cat. no. B10246, 5 μl) from Beckman Coulter; APC anti-α_4_β_7_ (A4B7R1; cat. no. 051514AB, 5 μl) by the NIH Nonhuman Primate Reagent Resource (R24 OD010976, and NIAID contract HHSN272201300031C), and BV605 anti-CCR6 (G034E3; cat. no. 353420, 5 μl), BV650 anti-CXCR3 (G025H7; cat. no. 353730, 5 μl) from BioLegend for 30 min at room temperature. This was followed by permeabilization with FOX3-transcription buffer set (cat. no. 00-5523-00) from eBioscience according to the manufacturer’s recommendation and subsequent intracellular staining with the following: BV750 anti-TNF-α (MAB11; cat. no. 566359, 5 μl), BUV396 anti-IFN-γ (B27; cat. no. 563563, 5 μl), BV510 anti-GranB (GB11; cat. no. 563388, 5 μl), BV421 anti-IL-10 (JES3-9D7; cat. no. 564053, 5 μl), PE-CF594 anti-Ki67 (B56; cat. no. 567120, 5 μl) from BD Biosciences; and FITC anti-Perforin (pf-344; cat. no. 3465-7, 5 μl) from MABTECH for 30 min at room temperature. Flow cytometry acquisitions were performed on a FACSymphony A5 and examined using FACSDiva software (BD Biosciences).

### Frequencies and cytokine levels of macaque rectal mucosal NK cells and T cells following SAMT-247 and PMA-Ionomycin stimulation

NK/ILC and T-cell frequencies and cytokine levels were measured in macaque rectal mucosa. Freshly collected rectal biopsies were processed to single cells and cultured in R10 in the presence/or absence of SAMT-247 and/or PMA-Ionomycin for 12 h. Subsequently, cells were stained for live cells with Live/Dead Blue dye (cat. no. L34962, 0.5 μl) from Thermo Fisher; followed by surface staining with the following: BUV737 anti-CD3 (SP34-2; cat. no. 741872, 5 μl), BV711 anti-CD4 (L200; cat. no. 563913, 5 μl), BV650 anti-NKp44 (P44-8; cat. no. 744302, 5 μl), Alexa 700 anti-CD20 (2H7; cat. no. 560631, 5 μl), BV786 anti-CD45 (D058-1283; cat. no. 563861, 5 μl) from BD Biosciences; APC-H7 anti-CD11b (ICRF44; cat. no. 47-0118-42, 5 μl), PE-Cy5 anti-CD95 (ICRF44; cat. no. 15-0959-42, 5 μl) from eBioscience; BV570 anti-CD8 (RPA-T8; cat. no. 301038, 5 μl), BV605 anti-CCR6 (G034E3; cat. no. 353420, 5 μl), APC anti-CXCR3 (G025H7; cat. no. 353708, 5 μl), from BioLegend and PE-Cy7 anti-NKG2A (Z199; cat. no. B10246, 5 μl) from Beckman Coulter for 30 min at room temperature. This was followed by permeabilization with a FOXP3-transcription buffer set (cat. no. 00-5523-00) from eBioscience according to the manufacturer’s recommendation and subsequent intracellular staining with the following: BV750 anti-TNF-α (MAB11; cat. no. 566359, 5 μl), BUV395 anti-IFN-γ (B27; cat. no. 563563, 5 μl), BV510 anti-GranB (GB11; cat. no. 563388, 5 μl), BV421 anti-IL-10 (JES3-9D7; cat. no. 564053, 5 μl) from BD Biosciences; PE-Cy5.5 anti-IL-17 (BL168; cat. no. 512314, 5 μl) from BioLegend; and FITC anti-Perforin (pf-344; cat. no. 3465-7, 5 μl) from MABTECH for 30 min at room temperature. Flow cytometry acquisitions were performed on a FACSymphony A5 and examined using FACSDiva software (BD Biosciences).

### Zinc intensity

NKG2A^+^ NK cells were isolated from cryopreserved healthy human PBMCs. NK cells were labelled with APC anti-NKG2A (Z199, cat. no. A60797) from Beckman Coulter and Aqua Live/Dead viability dye was used to exclude dead cells. After staining, cells were washed, passed through a 40 μm cell strainer, and sorted on an Astrios EQ flow cytometer. NKG2A^+^ of live cells were sorted with purity of 99%. Subsequently, NK cells were cultured in the presence or absence of SAMT-247 and/or PMA-Ionomycin stimulation for 7 h. Cells were plated on ibidi chamber slides. Cells were washed and treated with zinc chelator or remained untreated for 30 min according to the manufacturer recommendation using a cell-based zinc assay kit (cat. no. ab241014, Abcam). Subsequently, cells were washed and stained with zinc probe-green (cat. no. ab241014, Abcam) and DAPI (Molecular Probes) was used to visualize nuclei. Signals were visualized with a confocal laser-scanning microscope (Leica SP8, Leica Microsystems). Image processing was performed using the Imaris 9.2.1 software (Oxford Instruments).

### Statistical analysis

Statistical analysis was performed without testing normal distribution and equal variances of the data, and therefore non-parametric tests were used. The two-tailed Wilcoxon signed-rank test or two-tailed Mann–Whitney test was used to compare continuous factors between two paired or unpaired groups, respectively. Comparisons of differences between groups in the number of challenges before viral acquisition were assessed using the log-rank (Mantel–Cox) test of the discrete-time proportional hazards model. The average per-risk challenge of viral acquisition was estimated as the total number of observed infections divided by the number of administered challenges. Correlation analyses were performed using the non-parametric Spearman-rank correlation method with exact permutation and approximate two-tailed *P* values calculated for the number of pairs $\le$17 and >17, respectively. Since this research was conducted as exploratory, all *P* values are reported as nominal values without adjusting for multiple comparisons. No animals or data points were excluded from the analyses.

### Reporting summary

Further information on research design is available in the Nature Portfolio Reporting Summary linked to this article.

### Supplementary information

Reporting Summary

### Source data

Source Data Fig. 1 Raw data for Fig. 1.

Source Data Fig. 2 Raw data for Fig. 2.

Source Data Fig. 3 Raw data for Fig. 3.

Source Data Fig. 4 Raw data for Fig. 4.

Source Data Fig. 5 Raw data for Fig. 5.

Source Data Extended Data Fig. 1 Raw data for Extended Data Fig. 1.

Source Data Extended Data Fig. 2 Raw data for Extended Data Fig. 2.

Source Data Extended Data Fig. 3 Raw data for Extended Data Fig. 3.

Source Data Extended Data Fig. 4 Raw data for Extended Data Fig. 4.

Source Data Extended Data Fig. 5 Raw data for Extended Data Fig. 5.

Source Data Extended Data Fig. 6 Raw data for Extended Data Fig. 6.

Source Data Extended Data Fig. 7 Raw data for Extended Data Fig. 7.

Source Data Extended Data Fig. 8 Raw data for Extended Data Fig. 8.

Source Data Extended Data Fig. 9 Raw data for Extended Data Fig. 9.

Source Data Image file Raw data for Fig. 4a,b.

## Figures and Tables

**Extended Data Table 1:**
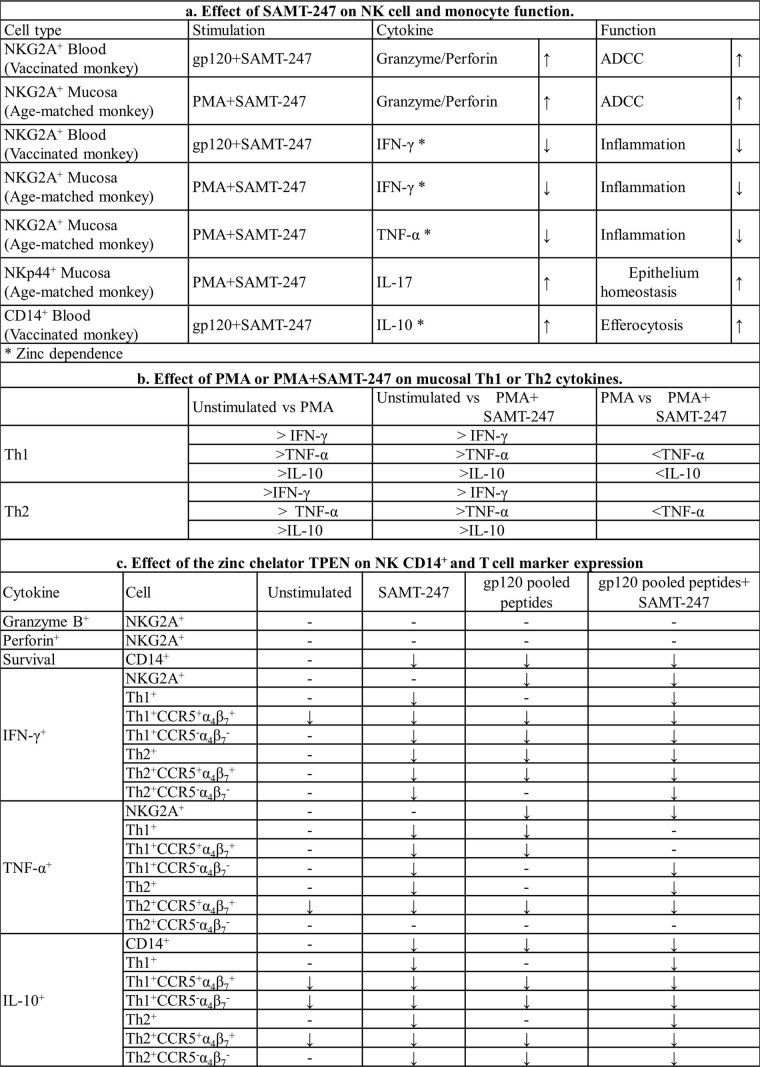
Effect of SMAT-247 and zinc chelator on different cell types in the presence or absence of different stimuli

**a**) Effect of SAMT-247 on NK cell and monocyte function.

**b**) Effect of PMA or PMA + SAMT-247 on mucosal Th1 or Th2 cytokines.

**c**) Effect of the zinc chelator TPEN on NK CD14^+^ and T cell marker expression

## Data Availability

All data in the manuscript and supplemental material are provided in the accompanying ‘Source Data’ files. Source data are provided with this paper.

## Code availability

N/A.
