# Publisher Correction: Vaccine plus microbicide effective in preventing vaginal SIV transmission in macaques

**DOI:** 10.1038/s41564-023-01412-z

**Published:** 2023-05-22

**Authors:** Mohammad Arif Rahman, Massimiliano Bissa, Isabela Silva de Castro, Sabrina Helmold Hait, James D. Stamos, Farzana Bhuyan, Ruth Hunegnaw, Sarkis Sarkis, Anna Gutowska, Melvin N. Doster, Ramona Moles, Tanya Hoang, Lisa M. Miller Jenkins, Ettore Appella, David J. Venzon, Hyoyoung Choo-Wosoba, Timothy Cardozo, Marc M. Baum, Daniel H. Appella, Marjorie Robert-Guroff, Genoveffa Franchini

**Affiliations:** 1 Animal Models and Retroviral Vaccines Section, National Cancer Institute, Bethesda, MD USA; 2 Section on Immune Biology of Retroviral Infection, National Cancer Institute, Bethesda, MD USA; 3 Translational Autoinflammatory Diseases Section, Laboratory of Clinical Immunology and Microbiology, National Institute of Allergy and Infectious Diseases, National Institutes of Health, Bethesda, MD USA; 4 Collaborative Protein Technology Resource, Laboratory of Cell Biology, National Cancer Institute, Bethesda, MD USA; 5 Chemical Immunology Section, National Cancer Institute, Bethesda, MD USA; 6 Biostatistics and Data Management Section, Center for Cancer Research, National Cancer Institute, Bethesda, MD USA; 7 New York University School of Medicine, NYU Langone Health, New York, NY USA; 8 Oak Crest Institute of Science, Monrovia, CA USA; 9 Synthetic Bioactive Molecules Section, National Institute of Diabetes and Digestive and Kidney Diseases, National Institutes of Health, Bethesda, MD USA

**Keywords:** HIV infections, Vaccines, Mucosal immunology, Innate immunity

Correction to: *Nature Microbiology* 10.1038/s41564-023-01353-7. Published online 6 April 2023.

In the version of this article initially published, the author list did not display first names. The list has been revised in the HTML and PDF versions of the article.

