# Supplementary material for: Vaccine plus microbicide effective in preventing vaginal SIV transmission in macaques
Source: Nat Microbiol. 2023 Apr 6;8(5):905–18. doi: 10.1038/s41564-023-01353-7 (PMC10159859; doi:10.1038/s41564-023-01353-7)
Supplement: Supplementary file 1 — Reporting Summary [file 41564_2023_1353_MOESM1_ESM.pdf]

nature portfolio

Corresponding author(s): Genoveffa Franchini

Last updated by author(s): Feb 15, 2023

Reporting Summary

Nature Portfolio wishes to improve the reproducibility of the work that we publish. This form provides structure for consistency and transparency in reporting. For further information on Nature Portfolio policies, see our Editorial Policies and the Editorial Policy Checklist.

Statistics

For all statistical analyses, confirm that the following items are present in the figure legend, table legend, main text, or Methods section.

n/a	Confirmed	
☐	☒	The exact sample size (*n*) for each experimental group/condition, given as a discrete number and unit of measurement
☐	☒	A statement on whether measurements were taken from distinct samples or whether the same sample was measured repeatedly
☐	☒	The statistical test(s) used AND whether they are one- or two-sided<br>*Only common tests should be described solely by name; describe more complex techniques in the Methods section.*
☐	☒	A description of all covariates tested
☐	☒	A description of any assumptions or corrections, such as tests of normality and adjustment for multiple comparisons
☐	☒	A full description of the statistical parameters including central tendency (e.g. means) or other basic estimates (e.g. regression coefficient) AND variation (e.g. standard deviation) or associated estimates of uncertainty (e.g. confidence intervals)
☐	☒	For null hypothesis testing, the test statistic (e.g. *F*, *t*, *r*) with confidence intervals, effect sizes, degrees of freedom and *P* value noted<br>*Give P values as exact values whenever suitable.*
☒	☐	For Bayesian analysis, information on the choice of priors and Markov chain Monte Carlo settings
☐	☒	For hierarchical and complex designs, identification of the appropriate level for tests and full reporting of outcomes
☐	☒	Estimates of effect sizes (e.g. Cohen's *d*, Pearson's *r*), indicating how they were calculated

Our web collection on statistics for biologists contains articles on many of the points above.

Software and code

Policy information about availability of computer code

Data collection	FACSymphony A5 and examined using FACSDiva software (BD Biosciences).<br>Confocal laser-scanning microscope (Leica SP8, Leica Microsystems, Buffalo Grove, Illinois, USA)<br>Molecular Devices E-max plate reader (San Jose, California, USA)<br>Droplet digital PCR
Data analysis	FlowJo software 10.6, Graph pad Prism 8.0, Imaris 9.2.1,

For manuscripts utilizing custom algorithms or software that are central to the research but not yet described in published literature, software must be made available to editors and reviewers. We strongly encourage code deposition in a community repository (e.g. GitHub). See the Nature Portfolio guidelines for submitting code & software for further information.

Data

Policy information about availability of data

All manuscripts must include a data availability statement. This statement should provide the following information, where applicable:

- Accession codes, unique identifiers, or web links for publicly available datasets
- A description of any restrictions on data availability
- For clinical datasets or third party data, please ensure that the statement adheres to our policy

All data are provided in the manuscript and supplemental material are provided in the “Source Data” file.

Human research participants

Policy information about studies involving human research participants and Sex and Gender in Research.

Reporting on sex and gender	Human PBMC were collected from healthy donor. The data regarding sex and gender were not shared with the authors.
Population characteristics	The authors don't have access to the data.
Recruitment	The authors were blinded regarding the recruitment of the healthy human donors. The donors were recruited by NIH blood bank.
Ethics oversight	Human PBMCs were obtained from eight healthy donors on an IRB-approved NIH protocol (99-CC-0168). Research blood donors provided written informed consent and blood samples were de-identified prior to distribution." Clinical Trials Number: NCT00001846.

Note that full information on the approval of the study protocol must also be provided in the manuscript.

Field-specific reporting

Please select the one below that is the best fit for your research. If you are not sure, read the appropriate sections before making your selection.

☒ Life sciences ☐ Behavioural & social sciences ☐ Ecological, evolutionary & environmental sciences

For a reference copy of the document with all sections, see nature.com/documents/nr-reporting-summary-flat.pdf

Life sciences study design

All studies must disclose on these points even when the disclosure is negative.

Sample size	Sample size was determined via consultation with our statistician. 50 macaques were divided into four groups as follows: I: 20 macaques; vaccine-microbicide group II: 18 macaques; vaccine only group III: 6 macaques; microbicide only group IV: 6 macaques control group.<br><br>Group sizes were determined based on a previous vaccine study comparing 14 vaccinated macaques and 18 controls, and on a pilot experiment in which we defined a time between microbicide administration and viral challenge which would decrease the microbicide efficacy so that we could observe an additive/Synergistic effect.<br><br>Historical control macaques and microbicide macaques were also used in statistical analysis as indicated. Infection rates were assumed to equal the previously observed gel only and microbicide groups, 0.333 and 0.133, respectively, and rates of 0.120 and 0.040 in the vaccine and combination groups. The last three are expected treatment efficacies of 60%, 64%, and 88%, relative to the gel only group. If the animals are given up to 14 viral challenges and the numbers of challenges to infection are compared using the Wald test of the proportional hazards model with all four groups analyzed together, then the power of the test at the two-sided 0.05 level between the vaccine group and the combination group was expected to be 79%, while the comparisons of these two groups individually vs the control group will have 82% and 99% power. The test of the microbicide group vs the combination group will have 63% power.
Data exclusions	No data were excluded.
Replication	The nature of the samples analyzed in the present studies, the limited amount of each sample collected from each animal and the cost of the non-human primate studies do not allow us to replicate the experiments. In the reported assays the replicates are represented by each animal enrolled in the study. All the data have been obtained with validated assays that have been used in previous publish work.
Randomization	50 macaques were randomized into 4 groups based on their weight, age and gender: Vaccine/microbicide group (20 macaques), Vaccine only group (n=18), microbicide only group (n=6), and control group (n=6).<br>When samples were available we performed experiment using all the samples.<br>For other experiments where samples were limited, samples were picked randomly from each group.<br>For each in vitro study, samples from each animals received all the different stimulation.
Blinding	The animal handlers were blinded to the vaccine groups. Always as possible, the investigators were blinded for in vitro assays. The data analyzes were unblinded.<br>The authors compared the immune responses between different group of animals and for that unblinded was necessary.<br>All the in vitro study was performed blinded. During analysis of data, such as correlation and comparison of different group response, the data were unblinded.

Reporting for specific materials, systems and methods

We require information from authors about some types of materials, experimental systems and methods used in many studies. Here, indicate whether each material, system or method listed is relevant to your study. If you are not sure if a list item applies to your research, read the appropriate section before selecting a response.

Materials & experimental systems

n/a	Involved in the study
☐	☒ Antibodies
☒	☐ Eukaryotic cell lines
☒	☐ Palaeontology and archaeology
☐	☒ Animals and other organisms
☒	☐ Clinical data
☒	☐ Dual use research of concern

Methods

n/a	Involved in the study
☒	☐ ChIP-seq
☐	☒ Flow cytometry
☒	☐ MRI-based neuroimaging

Antibodies

Antibodies used

Rectal mucosal NK/ILC phenotyping and cytokine expression upon gp120 peptides/PMA stimulation in vaccinated animals.
Live/Dead Aqua Dye (cat. #L34966, 0.5 µl) from Thermo Fisher
Alexa 700 anti-CD3 (SP34-2; cat. #557917, 5µl), Alexa 700 anti-CD20 (2H7; cat. #560631, 5µl), Alexa 700 anti-CD11b (ICRF44; cat. #557918, 5µl), APC-Cy7 anti-CD16 (3G8; cat. #557758, 5µl), PE-CF594 anti-CD56 (B159; cat. #562289, 5µl) BV650 anti-NKp44 (P44-8; cat. #744302, 5µl), BV786 anti-CD45 (D058-1283; cat. #563861,5 µl) from BD Biosciences (San Jose, California, USA); and PE-Cy7 anti-NKG2A (Z199; cat. # B10246, 5µl) from Beckman Coulter (Brea, California, USA) ;FOX3-transcription buffer set (cat. #00-5523-00) from eBioscience (San Diego, California, USA); BV421 anti-IFN-γ (B27; cat. #562988, 5µl) from BD Biosciences and PE-Cy5.5 anti-IL-17 (BL168; cat. # 512314, 5µl) from BioLegend (San Diego, California, USA)

CD4+ T-cell phenotypes
LIVE/DEAD™ Fixable Blue Dead Cell Stain (cat. #L23105, Thermo Fisher); Alexa 700 anti-CD3 (SP34-2; cat. #557917, 5µl), BV785 anti-CD4 (L200; cat. #563914, 5µl), PeCy5 anti-CD95 (DX2; cat. #559773, 5µl), BV650 anti-CCR5 (3A9; cat. #564999, 5µl), BUV496 anti-CD8 (RPA-T8; cat. #564804, 5µl), BUV737 anti-CD28 (CD28.2; cat. #612815, 5µl) and FITC anti-Ki67 (B56; cat. #556026, 5µl) from BD Biosciences; APC Cy7 anti-CXCR3 (G025H7; cat. #353722, 5µl), BV605 anti-CCR6 (G034E3; cat. #353420, 5µl), BV510 anti-CD127 (A019D5; cat. #351332, 5µl), BV750 anti-PD-1 (EH12.2H7; cat. #329965, 5µl) and BV711 anti-CD25 (BC96; cat. #302636, 5µl) from BioLegend; PE-eFluor 610 anti-CXCR5 (MU5UBEE; cat. #61-9185-42, 5µl), eFluor 450 anti-FoxP3 (236A/E7; cat. #48-4777-42, 5µl) from eBioscience; and APC anti-α4β7, provided by the NIH Nonhuman Primate Reagent Resource (R24 OD010976, and NIAID contract HHSN272201300031C).

Intracellular cytokines of Human blood NK cells using SAMT-247 and PMA stimulation with or without zinc chelator.
BUV737 anti-CD3 (SP34-2; cat. #741872, 5µl), Alexa700 anti-CD20 (2H7; cat. #560631, 5µl), BV786 anti-CD45 (HI30; cat. #563716, 5µl) from BD Biosciences; APC-H7 anti-CD11b (ICRF44; cat. #47-0118-42, 5µl) from eBioscience and PE-Cy7 anti-NKG2A (Z199; cat. # B10246, 5µl) from Beckman Coulter; FOX3-transcription buffer set (cat. #00-5523-00) from eBioscience; BV750 anti-TNF-α (MAB11; cat. #566359, 5µl), BUV396 anti-IFN-γ (B27; cat. #563563, 5µl), BV510 anti-GranB (GB11; cat. #563388, 5µl) from BD Biosciences; and FITC anti-Perforin (pf-344; cat. #3465-7, 5µl) from MABTECH

AIM assay
CD40 blocking antibody (HB14, cat. # 130-094-133, 5µl) from Miltenyi; CD49a (9F10, cat. # 555501, 2µl) and CD28 (CD28.2, cat. # 567117, 2µl) from BD Bioscience; LIVE/DEAD™ Fixable Blue Dead Cell Stain (cat. #L23105, Thermo Fisher); BV786 anti-CD45 (D058-1283; cat. # 563861, 5µl), BUV737 anti-CD3 (SP34-2; cat. # 741872, 5µl), BV711 anti-CD4 (L200; cat. # 740807, 5µl), BUV496 anti-CD8 (RPA-T8; cat. # 612942, 5µl), PE-CF594 anti-PDL1 (MIH1; cat. # 563742, 5µl), BB700 anti-CTLA-4 (BNI3; cat. # 566901, 5µl), PE-Cy5 anti- OX40 (CD134) (ACT35; cat. # 551500, 5µl), BUV563 anti-CD40L (CD154) (24-31; cat. # 752854, 5µl), PE-Cy7 anti- CD69 (FN50; cat. # 557745, 5µl), PE anti- CD95 (DX2; cat. # 555674, 5µl) from BD Bioscience; FITC anti-LAG3 (3DS223H; cat. #369326, 5µl) from Thermofisher; BV750 anti-PD1 (EH12.2H7; cat. # 329966, 5µl), Alexa700 anti-CXCR3 (G025H7; cat. # 353742, 5µl), BV605 anti-CCR6 (G034E3; cat. #353420, 5µl) from Biolegend; FOX3-transcription buffer set (cat. #00-5523-00) from eBioscience (San Diego, California, USA); BV510 anti-Ki67 (B56; cat. # 563462, 5µl) from BD Biosciences

Intracellular cytokines of macaque blood NK cells and T-cells using SAMT-247 and PMA stimulation with or without zinc chelator.
Live/Dead Blue dye (cat. #L34962, 0.5µl) from Thermo Fisher; followed by surface staining with the following: PE anti-CD45 (D058-1283; cat. #552833, 5µl), BB700 anti-CD3 (RPA-T8; cat. #566452, 5µl), Alexa 700 anti-CD3 (SP34-2; cat. #557917, 5µl), BV711 anti-CD4 (L200; cat. #563913, 5µl), BV786 anti-CCR5 (3A9; cat. #565001, 5µl), BUV737 anti-CD20 (2H7; cat. #612848, 5µl), BUV496 anti-CD16 (3G8; cat. #612944, 5µl), BUV661 anti-HLA-DR (G46-6; cat. #612980, 5µl), BUV805 anti-CD14 (M5E2; cat. #565779, 5µl) from BD Biosciences; APC-H7 anti-CD11b (ICRF44; cat. #47-0118-42, 5µl), PE-Cy5 anti-CD95 (DX2; cat. #15-0959-42, 5µl) from eBioscience; PE-Cy7 anti-NKG2A (Z199; cat. # B10246, 5µl) from Beckman Coulter; APC anti-α4β7 (A4B7R1; cat. # 051514AB, 5µl) by the NIH Nonhuman Primate Reagent Resource (R24 OD010976, and NIAID contract HHSN272201300031C), and BV605 anti-CCR6 (G034E3; cat. # 353420, 5µl), BV650 anti-CXCR3 (G025H7; cat. # 353730, 5µl) from BioLegend; FOX3-transcription buffer set (cat. #00-5523-00) from eBioscience; BV750 anti-TNF-α (MAB11; cat. #566359, 5µl), BUV396 anti-IFN-γ (B27; cat. #563563, 5µl), BV510 anti-GranB (GB11; cat. #563388, 5µl), BV421 anti-IL-10 (JES3-9D7; cat. #564053, 5µl), PE-CF594 anti-Ki67 (B56; cat. #567120, 5 µl) from BD Biosciences; and FITC anti-Perforin (pf-344; cat. #3465-7, 5 µl) from MABTECH

Frequencies and cytokine levels of macaque rectal mucosal NK cells and T-cells following SAMT-247 and PMA stimulation
Live/Dead Blue dye (cat. #L34962, 0.5µl) from Thermo Fisher; followed by surface staining with the following: BUV737 anti-CD3 (SP34-2; cat. #741872, 5µl), BV711 anti-CD4 (L200; cat. #563913, 5µl), BV650 anti-NKp44 (P44-8; cat. #744302, 5µl), Alexa700 anti-CD20 (2H7; cat. #560631, 5µl), BV786 anti-CD45 (D058-1283; cat. #563861, 5µl) from BD Biosciences; APC-H7 anti-CD11b (ICRF44; cat. #47-0118-42, 5µl), PE-Cy5 anti-CD95 (ICRF44; cat. #15-0959-42, 5µl) from eBioscience; BV570 anti-CD8 (RPA-T8; cat. #301038, 5µl), BV605 anti-CCR6 (G034E3; cat. #353420, 5µl), APC anti-CXCR3 (G025H7; cat. #353708, 5µl), from BioLegend and PE-Cy7 anti-

NKG2A (Z199; cat. # B10246, 5μl) from Beckman Coulter, FOX3-transcription buffer set (cat. #00-5523-00) from eBioscience; BV750 anti-TNF-α (MAB11; cat. #566359, 5μl), BUV395 anti-IFN-γ (B27; cat. #563563, 5μl), BV510 anti-GranB (GB11; cat. #563388, 5μl), BV421 anti-IL-10 (JES3-9D7; cat. # 564053, 5μl) from BD Biosciences; PE-Cy5.5 anti-IL-17 (BL168; cat. # 512314, 5μl) from BioLegend; and FITC anti-Perforin (pf-344; cat. #3465-7, 5μl) from MABTECH

Validation

Marker Clone Validated website
1 CD40 HB14 https://www.nhpreagents.org/ReactivityDatabase
2 CD49a 9F10 https://www.bdbiosciences.com/en-us/products/reagents/flow-cytometry-reagents/research-reagents/single-color-antibodies-ruo/pe-cy-5-mouse-anti-human-cd49d.559880
3 CD45 D058-1283 https://www.bdbiosciences.com/en-us/products/reagents/flow-cytometry-reagents/research-reagents/single-color-antibodies-ruo/pe-mouse-anti-nhp-cd45.552833
4 CD3 SP34-2 https://www.citeab.com/antibodies/2412955-557757-bd-pharmingen-apc-cy-7-mouse-anti-human-cd3?des=080d3bb9991f1653
5 CD20 2H7 https://www.bdbiosciences.com/en-us/products/reagents/flow-cytometry-reagents/research-reagents/single-color-antibodies-ruo/pe-cf594-mouse-anti-human-cd20.562295
6 CD11b ICRF44 https://www.thermofisher.com/us/en/home/life-science/cell-analysis/cell-analysis-learning-center/cell-analysis-resource-library/ebioscience-resources/human-antibody-cross-reactivity-chart.html
7 CD16 3G8 https://www.thermofisher.com/us/en/home/life-science/cell-analysis/cell-analysis-learning-center/cell-analysis-resource-library/ebioscience-resources/human-antibody-cross-reactivity-chart.html
8 CD56 B159 https://www.nhpreagents.org/ReactivityDatabase
9 NKp44 P44-8 https://www.biolegend.com/en-us/products/apc-anti-human-cd336-nkp44-antibody-3850
10 NKG2A Z199 https://www.beckman.com/reagents/coulter-flow-cytometry/antibodies-and-kits/single-color-antibodies/cd159a/a60797
11 CCR5 3A9 https://www.bdbiosciences.com/en-us/products/reagents/flow-cytometry-reagents/research-reagents/single-color-antibodies-ruo/purified-mouse-anti-human-cd195.556041
12 CD28 CD28.2 https://www.bdbiosciences.com/en-us/products/reagents/functional-cell-based-reagents/purified-na-le-mouse-anti-human-cd28.567117
13 CD4 L200 https://www.bdbiosciences.com/en-us/products/reagents/flow-cytometry-reagents/research-reagents/single-color-antibodies-ruo/fitc-mouse-anti-human-cd4.550628
14 CD8 RPA-T8 https://www.thermofisher.com/us/en/home/life-science/cell-analysis/cell-analysis-learning-center/cell-analysis-resource-library/ebioscience-resources/human-antibody-cross-reactivity-chart.html
15 CD127 A019D5 https://www.citeab.com/antibodies/524313-351304-pe-anti-human-cd127-il-7r-antibody
16 PD1 EH12.1 https://www.nature.com/articles/s41586-020-03041-6
17 HLA-DR G46-6 https://www.fishersci.com/shop/products/hla-dr-mouse-anti-human-rhesus-cynomolgus-baboon-r718-clone-g46-6-also-known-as-l243-bd-horizon/p-7227060
18 CTLA-4 BNI3 https://www.citeab.com/antibodies/2408361-555851-bd-pharmingen-purified-mouse-anti-human-cd15?des=e3d22b3bfa1d8952
19 PDL1 EH12.2H7 https://www.citeab.com/antibodies/523807-329902-purified-anti-human-cd279-pd-1-antibody?des=7156e11a58406a10
20 OX40 (CD134) ACT35 https://www.abcam.com/recombinant-rhesus-monkey-cd134--ox40l-receptor-protein-active-ab221322.html?productWallTab=ShowAll
21 CD25 BC96 https://www.thermofisher.com/us/en/home/life-science/cell-analysis/cell-analysis-learning-center/cell-analysis-resource-library/ebioscience-resources/human-antibody-cross-reactivity-chart.html
22 CXCR5 MU5UBEE https://www.thermofisher.com/us/en/home/life-science/cell-analysis/cell-analysis-learning-center/cell-analysis-resource-library/ebioscience-resources/human-antibody-cross-reactivity-chart.html
23 α4β7 A4B7R1 https://www.nhpreagents.org/Store/CategoryID/1/ProductID/37
24 Ki67 B56 https://www.nhpreagents.org/ReactivityDatabase
25 IL-17 BL168 https://www.nature.com/articles/s41564-020-00841-4
26 FoxP3 236A/E7 https://www.thermofisher.com/us/en/home/life-science/cell-analysis/cell-analysis-learning-center/cell-analysis-resource-library/ebioscience-resources/human-antibody-cross-reactivity-chart.html
27 Perforin pf-344 https://www.mabtech.com/products/anti-human-perforin-antibody-pf-344-fitc-3465-7-0
28 GranB GB11 https://www.nhpreagents.org/ReactivityDatabase
29 IL-10 JES3-9D7 https://www.nhpreagents.org/ReactivityDatabase
30 CD69 FN50 https://www.thermofisher.com/us/en/home/life-science/cell-analysis/cell-analysis-learning-center/cell-analysis-resource-library/ebioscience-resources/human-antibody-cross-reactivity-chart.html
31 CD95 DX2 https://www.thermofisher.com/us/en/home/life-science/cell-analysis/cell-analysis-learning-center/cell-analysis-resource-library/ebioscience-resources/human-antibody-cross-reactivity-chart.html
32 CD40L (CD154) 24-31 https://www.nhpreagents.org/ReactivityDatabase
33 CXCR3 G025H7 https://www.biolegend.com/en-us/products/biotin-anti-human-cd183-cxcr3-antibody-13320
34 CCR6 G034E3 https://www.biolegend.com/en-us/products/purified-anti-human-cd196-ccr6-antibody-7512
35 LAG3 3DS223H https://www.citeab.com/antibodies/3246730-17-2239-42-cd223-lag-3-monoclonal-antibody-3ds223
36 IFN-γ B27 https://www.bdbiosciences.com/en-us/products/reagents/immunoassay-reagents/purified-mouse-anti-human-ifn.554699
37 CD14 M5E2 https://www.bdbiosciences.com/en-us/products/reagents/flow-cytometry-reagents/research-reagents/single-color-antibodies-ruo/apc-mouse-anti-human-cd14.561383
38 TNF-α MAB11 https://www.bdbiosciences.com/en-us/products/reagents/immunoassay-reagents/elisa/biotin-mouse-anti-human-tnf.554511

Animals and other research organisms

Policy information about studies involving animals; ARRIVE guidelines recommended for reporting animal research, and Sex and Gender in Research

Laboratory animals	Fifty female Indian rhesus macaques obtained from the free-range breeding colony on Morgan Island, South Carolina, were used in this study. The macaques, aged 2 to 3 years at study initiation.<br>One time rectal tissue samples from 9 female random rhesus macaque were collected for this project to do some in vitro study.
Wild animals	No wild animals were used in this study.
Reporting on sex	Fifty female Indian rhesus macaques were used in the study. It was necessary to use female macaques since the study tested a candidate vaccine in combination with vaginal gel containing SAMt-247 microbicide to understand the protective efficacy of the combination. the consideration of sex in this study design an interpretation of results are described throughout the text and in the on line method section.<br>One time rectal tissue samples from 9 female random rhesus macaque were collected for this project to do some in vitro study.
Field-collected samples	No field samples were collected in this study.
Ethics oversight	The NCI (National Cancer Institute) ACUC (Animal Care and Use Committee) approved and provided guidance for the animal protocol.<br>The 9 rectal tissue collection was approved by the Tulane Animal Care and Use Committee (ACUC).

Note that full information on the approval of the study protocol must also be provided in the manuscript.

Flow Cytometry

Plots

Confirm that:

☒ The axis labels state the marker and fluorochrome used (e.g. CD4-FITC).

☒ The axis scales are clearly visible. Include numbers along axes only for bottom left plot of group (a 'group' is an analysis of identical markers).

☒ All plots are contour plots with outliers or pseudocolor plots.

☒ A numerical value for number of cells or percentage (with statistics) is provided.

Methodology

Sample preparation	Sample preparation is described in "online methods" section.<br><br>Freshly collected rectal biopsies were digested with collagenase (2 mg/ml; Sigma-Aldrich) in the absence of FBS in 370 C for 1 hour, then it was mechanically separated by using a 10ml syringe with a blunt head cannula. It was washed with R10 and pass through 70um cell strainer. Single cells were counted and used for the experiment.<br><br>EDTA whole blood was layered on the top of Ficoll Plaque (GE Healthcare, Chicago, Illinois, USA), and centrifuged at 2800rmp, 30 minutes, accelerator 4 and decelerator 4. The white PBMC band was collected, washed with PBS and used for experiments.<br><br>Neutrophils were isolated following isolation of PBMCs by Ficoll Plaque (GE Healthcare, Chicago, Illinois, USA), the cellular pellet was added to an equal volume of 20% dextran in water, gently mixed, and incubated for 1 min. Approximately three volumes of PBS were added, mixed again, and incubated in the dark for 50-60 minutes. At the end of incubation, the clear layer at the top of the tube containing neutrophils was collected. Cells were pelleted and treated with ACK lysing buffer (Quality Biological, Gaithersburg, MD, USA) for 5 min at 37ºC, washed with R10 and counted.
Instrument	Cytometry acquisition was done using the FACS Symphony A5 or FACS LSRII.
Software	Acquisition was done using FACSDiva software (BD Biosciences, San Jose, California, USA). Cytometry data were analysed using Flow Jo LLC, V10.6
Cell population abundance	Human NKG2A+ cells were sort with 99% purity. A starting 20-30 million cryo preserved PBMC was used for the sorting and 2-3 million NKG2A+ cells were obtained.
Gating strategy	NK/ILC gating<br>Singlets, Live cells, CD45+, CD3-CD20-CD11b-, NKp44+/NKG2A+/NKp44-NKG2A-<br><br>Th1 and Th2<br>Gating was done on live CD3+CD4+ cells and on vaccine induced Ki67+ cells. CXCR3 and CCR6 expression were used to identify Th1 or Th2 populations. CXCR3-CCR6- is Th2 and CXCR3+CCR6- is Th1 cells

☒ Tick this box to confirm that a figure exemplifying the gating strategy is provided in the Supplementary Information.
