# Supplementary material for: Vaccine plus microbicide effective in preventing vaginal SIV transmission in macaques
Source: Nat Microbiol. 2023 Apr 6;8(5):905–18. doi: 10.1038/s41564-023-01353-7 (PMC10159859; doi:10.1038/s41564-023-01353-7)
Supplement: Supplementary file 16 — Raw data for Fig. 4a,b. [file 41564_2023_1353_MOESM16_ESM.pdf]

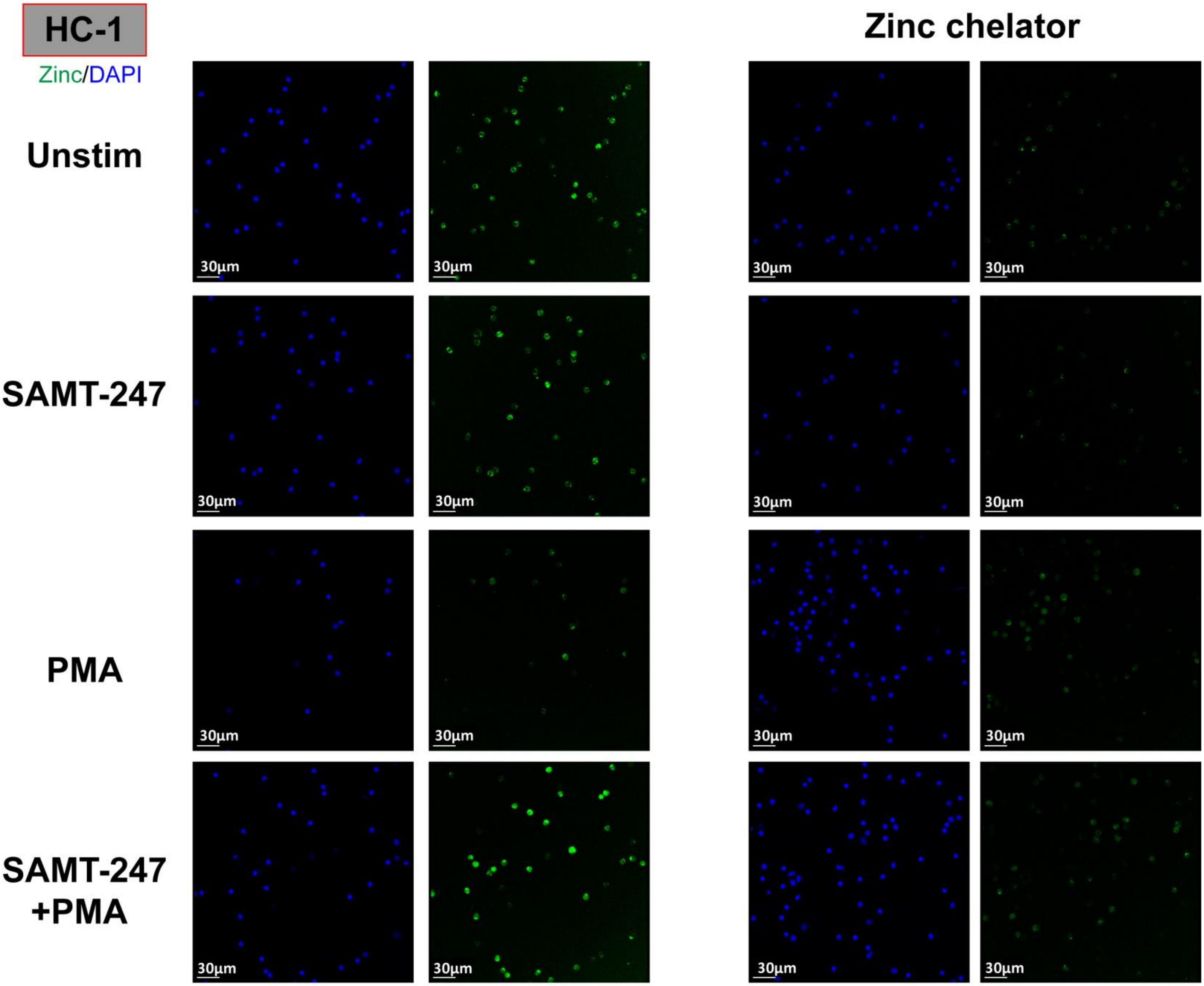
HC-1
Zinc/DAPI
Zinc chelator
Unstim
SAMT-247
PMA
SAMT-247
+PMA
30µm

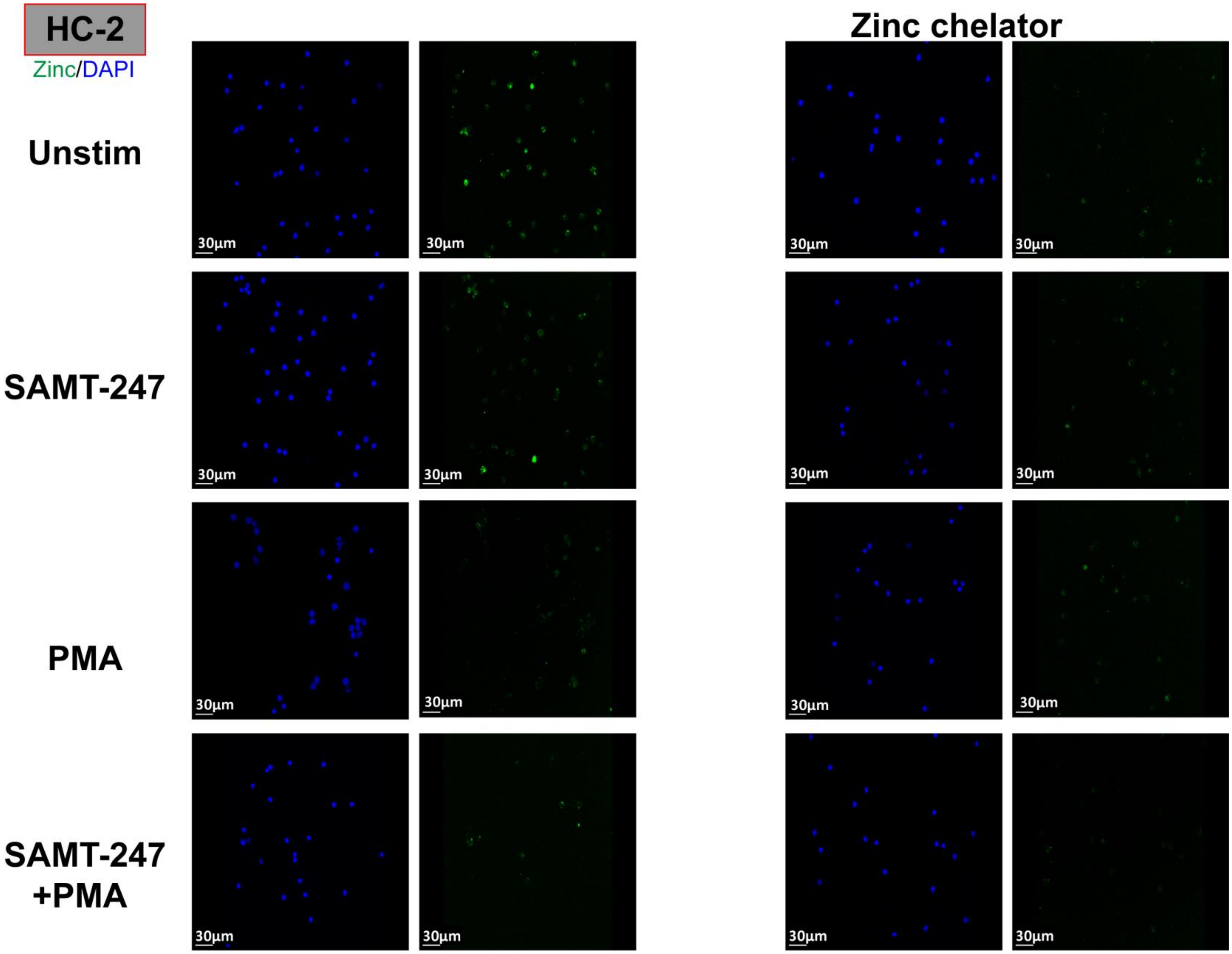

HC-2
Zinc/DAPI
Zinc chelator
Unstim
SAMT-247
PMA
SAMT-247
+PMA
30µm

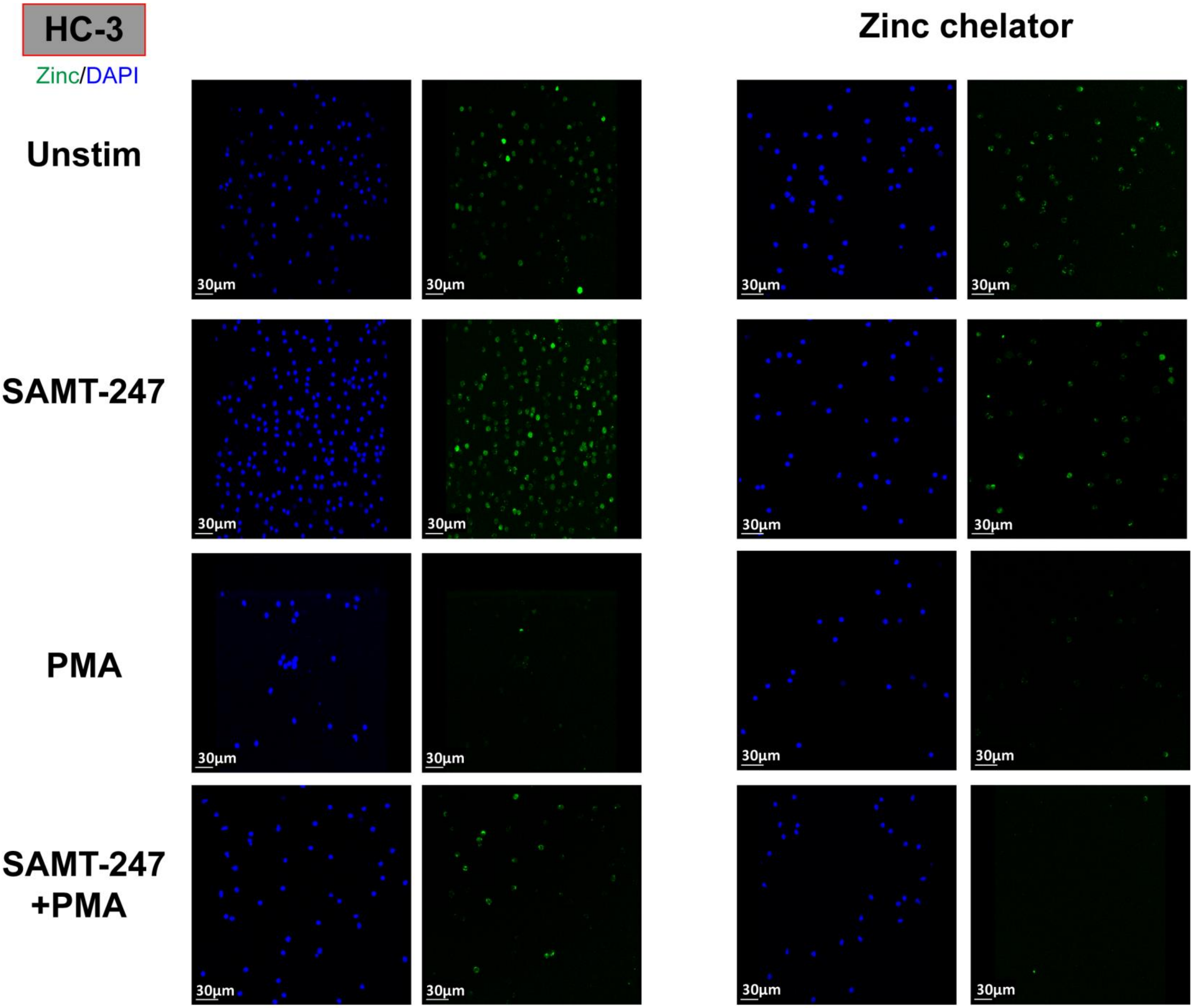

HC-3
Zinc/DAPI
Zinc chelator
Unstim
SAMT-247
PMA
SAMT-247 +PMA
30μm

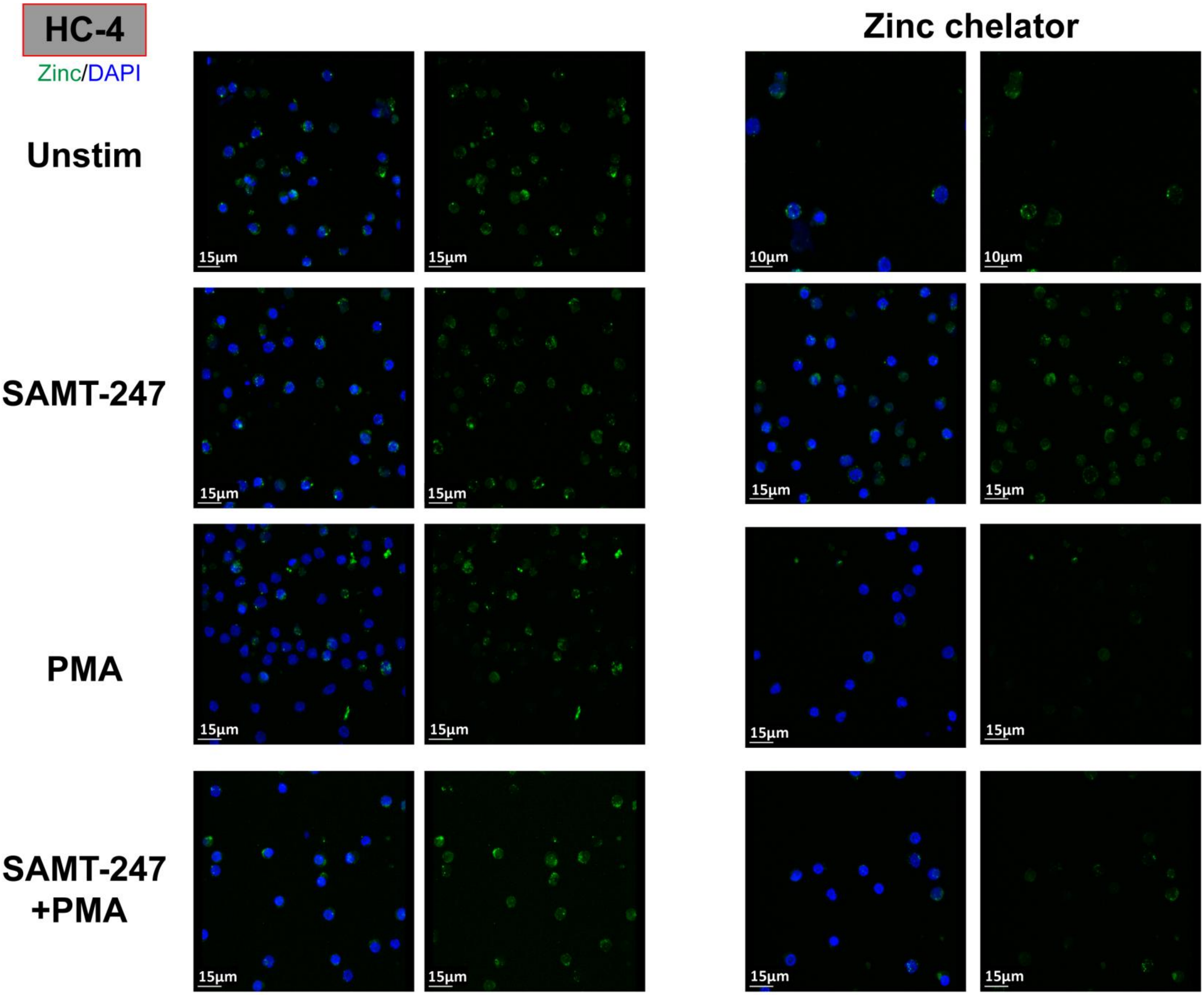

HC-4
Zinc/DAPI
Zinc chelator
Unstim
SAMT-247
PMA
SAMT-247 +PMA
15µm
10µm

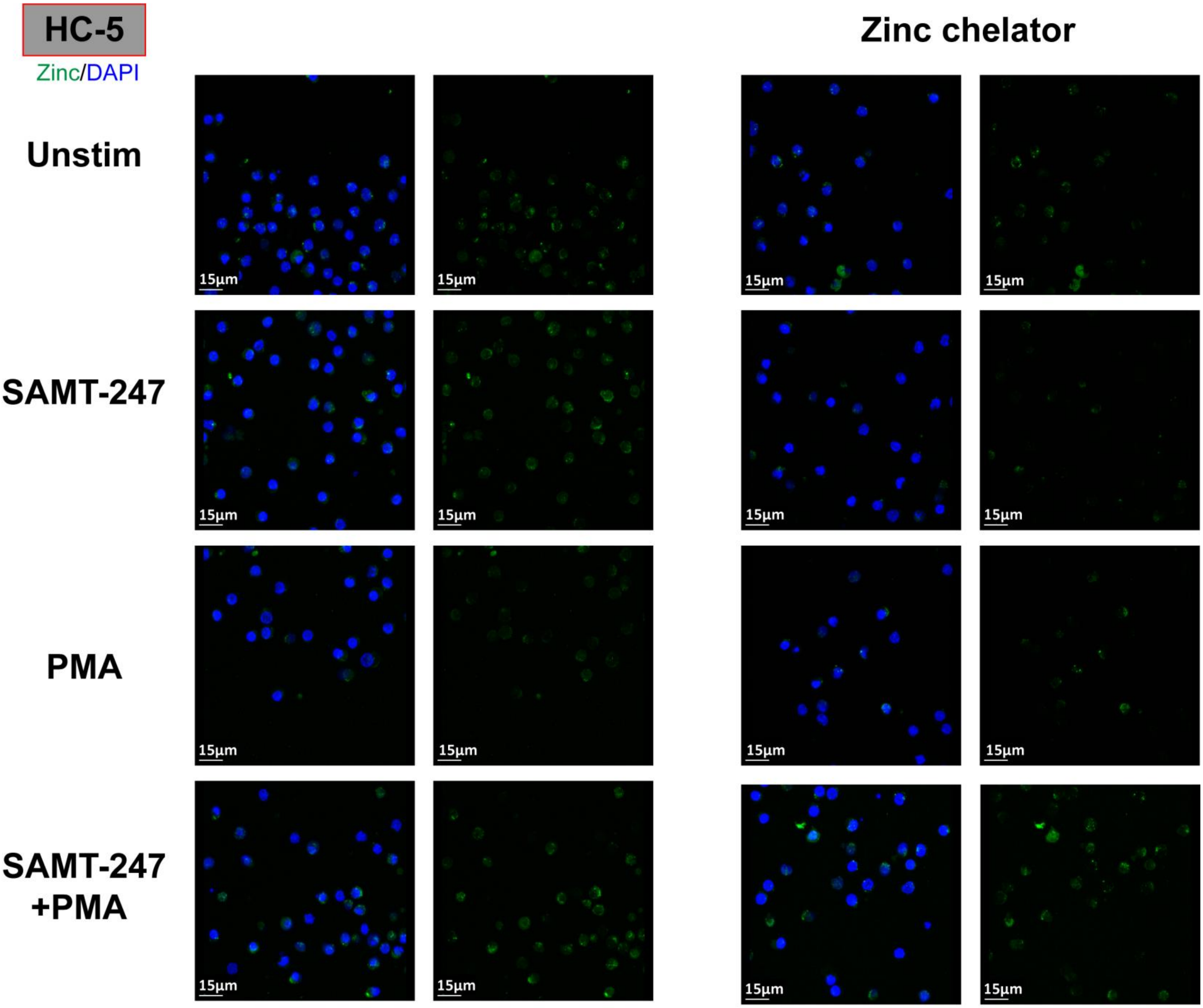
HC-5
Zinc/DAPI
Zinc chelator
Unstim
SAMT-247
PMA
SAMT-247
+PMA
15µm

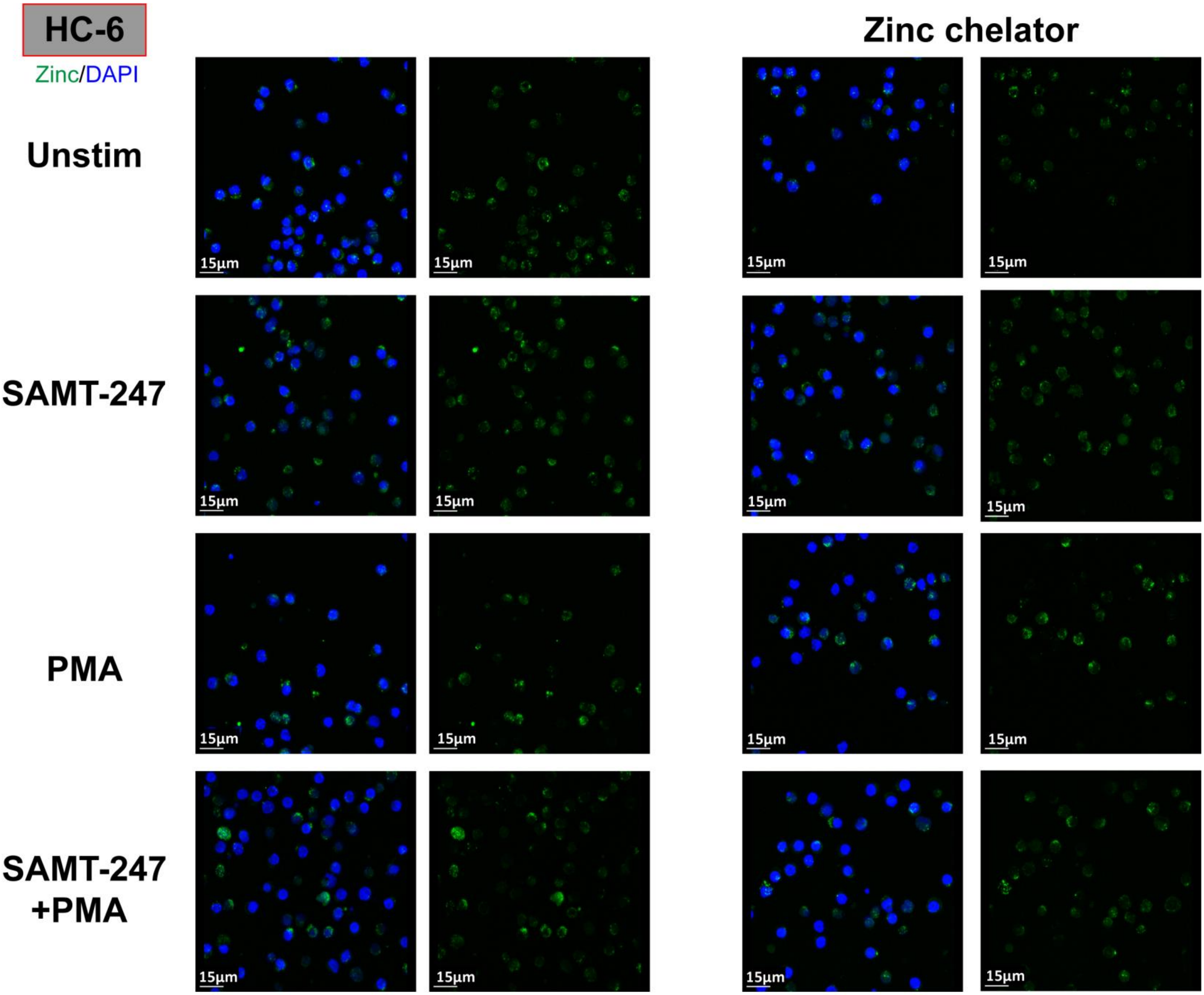
HC-6
Zinc/DAPI
Zinc chelator
Unstim
SAMT-247
PMA
SAMT-247
+PMA
15µm

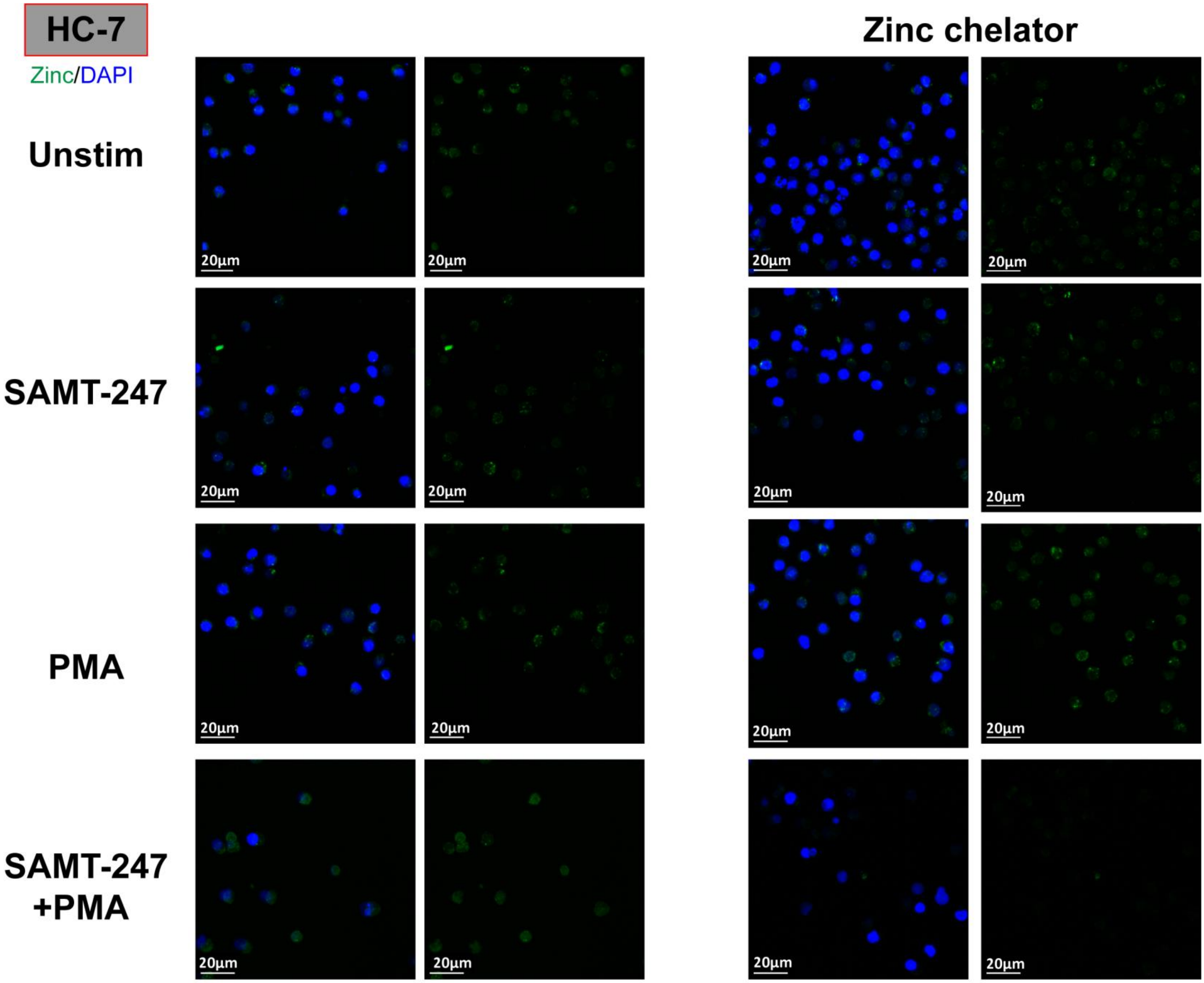
HC-7
Zinc/DAPI
Zinc chelator
Unstim
SAMT-247
PMA
SAMT-247 +PMA
20μm

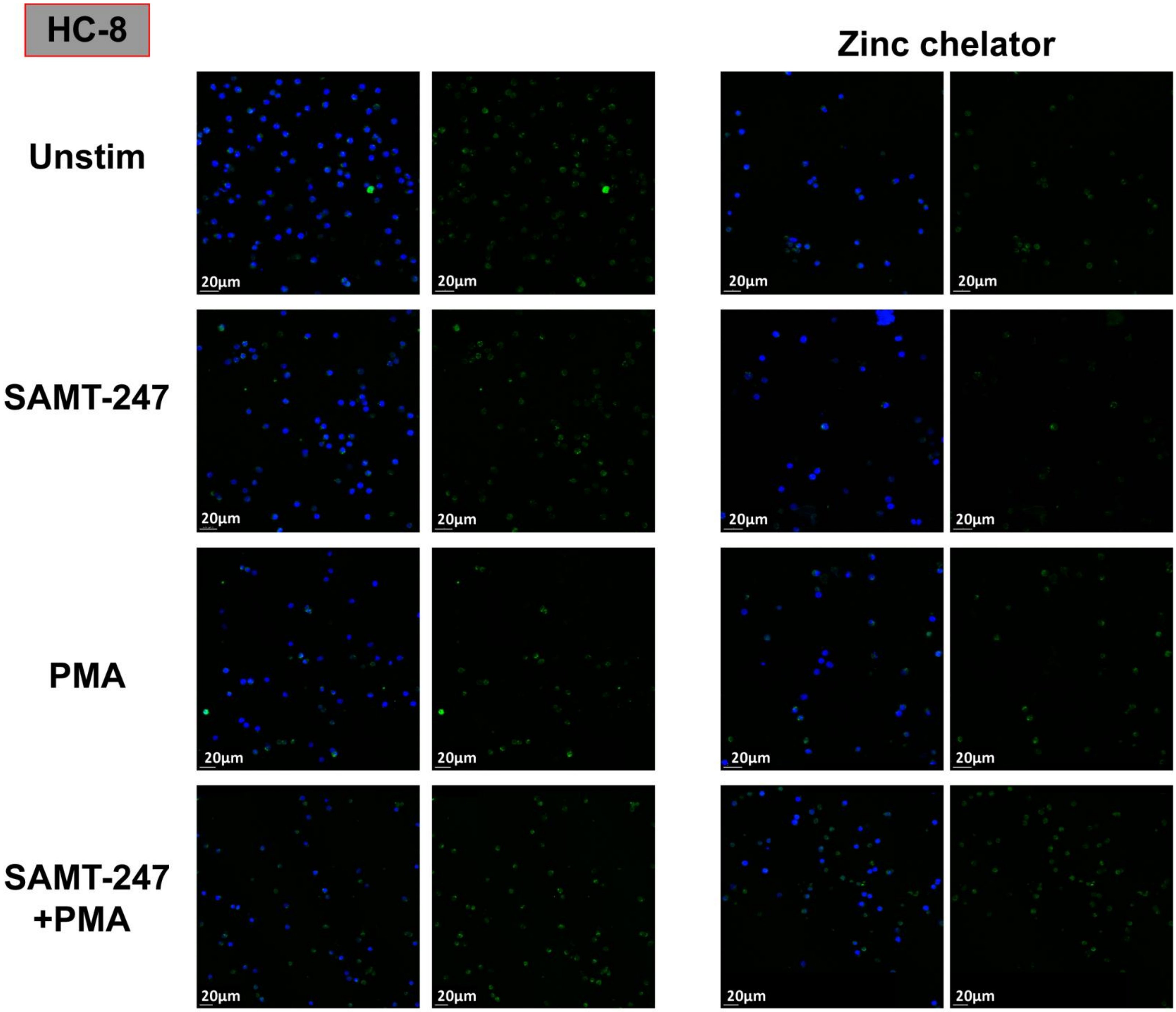

HC-8
Zinc chelator
Unstim
SAMT-247
PMA
SAMT-247
+PMA
20µm
